# PPAR**α**–NF-**κ**B heterodimer mediates obesity-induced diastolic dysfunction through autocrine production of IL-6

**DOI:** 10.1172/JCI196238

**Published:** 2026-02-12

**Authors:** Shin-ichi Oka, Eun-Ah Sung, Peiyong Zhai, Kevin B. Schesing, Santosh Bhat, Adave Chin, Jiyeon Park, Yeun-Jun Chung, Akihiro Shirakabe, Takanobu Yamamoto, Yoshiyuki Ikeda, Wataru Mizushima, Shohei Ikeda, Mingming Tong, Jaemin Byun, Michinari Nakamura, Samuel I. Kim, Jamie Francisco, Dominic P. Del Re, Junichi Sadoshima

**Affiliations:** 1 Rutgers New Jersey Medical School Department of Cell Biology and Molecular Medicine, Rutgers Biomedical and Health Sciences, Newark, New Jersey, USA.; 2 Precision Medicine Research Center, College of Medicine, The Catholic University of Korea, Seoul, Korea.

**Keywords:** Cardiology, Metabolism, Transcription

## Abstract

Obesity is accompanied by increases in free fatty acids (FFAs) in the systemic circulation, and patients with obesity often develop cardiac hypertrophy and diastolic dysfunction, termed obesity cardiomyopathy. Proinflammatory cytokines, including IL-6, have been implicated in the pathogenesis of the cardiac dysfunction associated with obesity cardiomyopathy. Elevation of FFAs induced by high-fat diet (HFD) consumption induced diastolic dysfunction in the heart as early as after 1 month. HFD consumption directly stimulated IL-6 production in cardiomyocytes before local inflammation developed and induced diastolic dysfunction even in the presence of macrophage depletion with clodronate in the heart. PPARα played an essential role in mediating *Il6* transcription in response to HFD consumption by forming a heterodimer with p50/RelA and binding to the NF-κB element in cardiomyocytes. Local production of IL-6 in cardiomyocytes, in turn, mediated the development of diastolic cardiac dysfunction. HFD-induced diastolic dysfunction was attenuated by cardiac-specific deletion of either *Pparα* or *Il6*, as well as by interference with the PPARα–NF-κB heterodimer formation by a molecular decoy. These results suggest elevated FFA levels directly upregulate *Il6* through the PPARα–NF-κB heterodimer in cardiomyocytes and highlight autocrine production of IL-6 as a key downstream mechanism in the initial development of diastolic dysfunction.

## Abstract 2

Direct stimulation of IL-6 transcription by PPARalpha in cardiomyocytes plays an importnat role in mediating the initial development of obesity cardiomyopathy.

## Graphical abstract

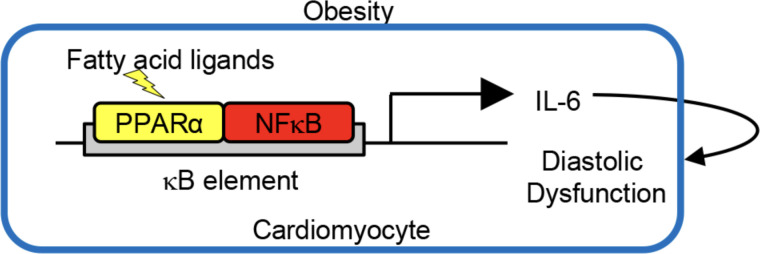

## Introduction

The prevalence of obesity, a major risk factor for type 2 diabetes, is increasing rapidly worldwide (1). More than half of patients with obesity develop myocardial dysfunction, known as obesity cardiomyopathy (2). In its early phase, diastolic dysfunction, left ventricular (LV) hypertrophy, and fibrosis are observed. Some patients develop heart failure with preserved ejection fraction (HFpEF), whereas others develop heart failure with reduced ejection fraction (3). Currently, there is no effective medical treatment for obesity cardiomyopathy and HFpEF (4).

The hearts of patients with obesity, insulin resistance, type 2 diabetes, and HFpEF often develop low-grade inflammation (5, 6). Pro-inflammatory cytokines and chemokines, including TNF-α, IL-1β, IL-6, and MCP-1, play an essential role in mediating the development of obesity cardiomyopathy (7, 8). Among the cytokines, IL-6 has been frequently associated with insulin resistance, diastolic dysfunction, fibrosis, and other features of obesity cardiomyopathy in humans (9) and in experimental animals fed a high-fat diet (HFD) (10, 11). IL-6 acts by binding to soluble IL-6 receptors and gp130, stimulating a trans-signaling response (10) and promoting cardiac fibrosis, hypertrophy, and stiffening through multiple mechanisms, including dephosphorylation of titin in cardiomyocytes (12). Importantly, however, which cell type produces the cytokines during the initial phase of obesity cardiomyopathy and how the cytokine production is induced are poorly understood.

NF-κB is a transcription factor composed of Rel and p50 proteins. NF-κB plays a central role in immune and inflammatory reactions. Although free fatty acid (FFA) activates the proinflammatory NF-κB pathway in the liver (13), the precise mechanism connecting FFA and NF-κB is not well understood. Importantly, many patients with mild obesity have either mild or even no obvious inflammation in the heart. It is important to elucidate how inflammation is initiated in the heart in these conditions.

PPARα is a nuclear receptor involved in fatty acid oxidation (14–17). The activity of PPARα is regulated by FFA ligands, PGC-1s, transcription (18), and posttranslational modification (17). PPARα is critically involved in the pathogenesis of obesity cardiomyopathy (19), and overexpression of PPARα in the heart induces lipotoxicity and obesity cardiomyopathy (20). Downregulation of PPARα in systemic *Ppara*-KO mice is generally protective against obesity cardiomyopathy (20–22). These observations suggest PPARα is a therapeutic target for obesity cardiomyopathy. However, whether PPARα inhibits or stimulates inflammation, an important mechanism facilitating the progression of obesity cardiomyopathy, has been controversial (23–26).

Because obesity is a condition in which fatty acid ligands are enriched in the body and induce low-grade inflammation (27), we reasoned that PPARα may mediate obesity-induced low-grade inflammation. We here propose that PPARα induces *Il6* in cardiomyocytes in obesity through physical interaction with NF-κB. We asked (a) whether upregulation of IL-6 in cardiomyocytes through a PPARα-dependent mechanism plays an important role in mediating the diastolic dysfunction in response to HFD consumption, and (b) whether heterodimerization between PPARα and an NF-κB subunit, and binding of the heterodimer to the κB element play an essential role in mediating IL-6 production. Our study provides mechanistic information about how obesity initiates local production of IL-6, which, in turn, leads to diastolic dysfunction.

## Results

### PPARα is activated during the development of diastolic dysfunction.

To induce diastolic dysfunction, we fed mice a HFD (60 kcal%), an established method of inducing obesity cardiomyopathy (28) (Supplemental Figure 1A; supplemental material available online with this article; https://doi.org/10.1172/JCI196238DS1). After 1 to 3 months of HFD consumption, diastolic dysfunction was assessed by performing pressure-volume (PV) loop analyses. The PV loop analysis continuously measures LV pressure and volume in the beating heart (Supplemental Figure 1B). Diastolic dysfunction is characterized by elevated LV blood pressure at end-diastole. We used end-diastolic pressure (EDP) and EDP-volume relationship (EDPVR) as indices of diastolic function (29). Diastolic dysfunction, characterized by increased EDP and EDPVR, was observed as early as after 1 month of HFD consumption and remained significant at 3 months in WT mice (Supplemental Figure 1C). Both male and female mice exhibited similar trends, and no significant sex-based differences were observed in either EDP or EDPVR measurements (Supplemental Figure 1C). Among genes known to be involved in diastolic dysfunction, *Atp2a2* was downregulated, and *Myh7* was upregulated after 1 month of HFD consumption (Supplemental Figure 1D). *Mmp2* showed a trend toward upregulation, whereas *Col1a1* expression was not significantly changed (Supplemental Figure 1D). *Atp2a2* contributes to myocardial relaxation via calcium reuptake, and its downregulation may contribute to diastolic dysfunction (30). *Myh7*, a fetal isoform of β-myosin heavy chain, is re-expressed under pathological conditions and is associated with impaired contractile and relaxation properties (31). *Mmp2* and *Col1a1* are involved in fibrosis (32), and their limited induction after 1 month of HFD is consistent with the absence of overt myocardial fibrosis at this early time point (Figure 2E). Cardiac systolic function, evaluated via the end-systolic PV relationship and the echocardiographically determined LV ejection fraction (LVEF), was preserved under HFD-consumption conditions (Supplemental Figure 1E). LV end-diastolic dimension was increased after 2 months and LV end-systolic dimension was increased after 3 months of HFD consumption (Supplemental Figure 1F), whereas LVEF was not altered at these time points. These results are consistent with the presence of volume overload during HFD consumption. It should be noted that both diastolic dysfunction and RNA expression of genes involved in diastolic/cardiac dysfunction were observed as early as after 1 month of HFD consumption. Thus, elevation of LVEDP is caused by diastolic dysfunction and not merely by volume overload.

**Figure 1:**
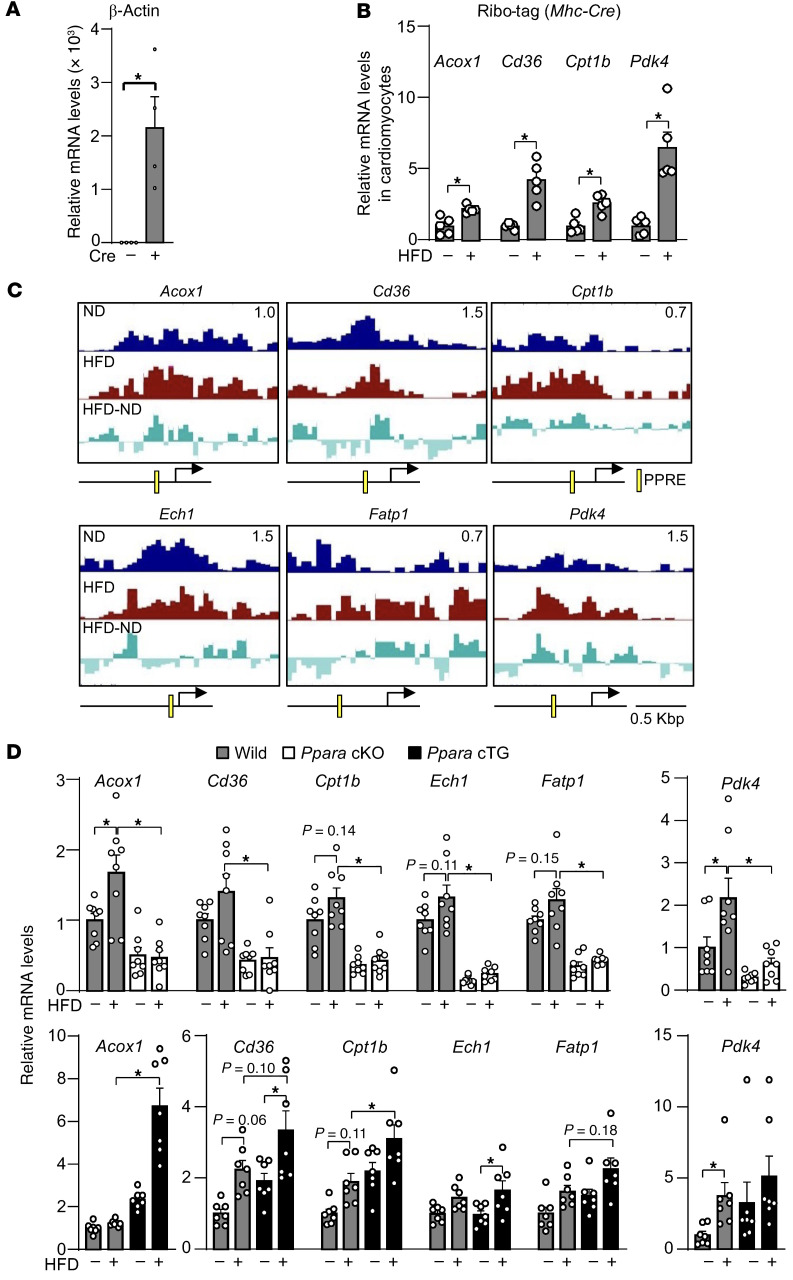
PPARα is functional under HFD-consumption conditions. (**A**) Successful mRNA isolation from cardiomyocyte-specific Ribo-Tag mice. (**B**) HFD induces PPAR target gene expression in cardiomyocytes in the heart in vivo. (**A** and **B**) Ribosome-associated RNA was isolated with HA-Rpl22, which is expressed in cardiomyocytes in a *Myh6*-Cre-dependent manner. Expression of indicated genes was examined. (**C**) PPARα localizes near PPRE in the promoters of its target genes, with heterogeneous binding responses to HFD consumption. ChIP-Seq was performed once using pooled heart samples from 3 mice after 1 month of HFD feeding. The *y* axis in the University of California, Santa Cruz, genome browser is indicated at top right (0.7–1.5) of each chart. Difference in PPARα occupancy between ND and HFD conditions is shown as ND-HFD. (**D**) HFD-induced PPARα target gene expression is inhibited in *Ppara-*cKO but enhanced in Tg-*Ppara* mice. Expression levels of indicated PPAR target genes after 1 month of HFD consumption were examined in *Ppara*-cKO and Tg-*Ppara* mice. The numbers of mice examined in each experimental group were as follows: 4 (**A**), 5 (**B**), and 7–8 (**C**). Statistical significance (*P* < 0.05) is indicated by an asterisk and was assessed using Student’s *t* test (**A** and **B**), the Kruskal-Wallis test for *Pdk4* (**C**), and ANOVA for other comparisons (**C**).

Because FFAs act as ligands for PPARα, increased plasma levels of FFAs resulting from HFD consumption may activate PPARα. To investigate whether HFD activates PPARα in cardiomyocytes, we used Ribo-tag mice — transgenic mice with Cre driver–dependent expression of HA-tagged Rpl22, a ribosomal protein — and evaluated whether known targets of PPARα are upregulated in response to HFD consumption. In this system, by crossing Ribo-tag mice with *Myh6*-Cre (Ribo-tag: *Myh6*-*Cre*) mice, HA-Rpl22 is expressed only in cardiomyocytes, such that immunoprecipitation with anti-HA antibody selectively pulls down ribosome-associated mRNA from cardiomyocytes. As shown in Figure 1A, after immunoprecipitation with anti-HA antibody, β*-actin* (*Actb*) mRNA was significantly enriched in heart samples from Ribo-tag: *Myh6*-Cre mice compared with in those from Ribo-tag mice, confirming Cre-dependent expression of HA-Rpl22 and successful isolation of cardiomyocyte-specific mRNA. The expression of PPARα target genes, including *Acox1*, *Cd36*, *Cpt1b*, and *Pdk4*, was significantly upregulated in cardiomyocytes in Ribo-tag: *Myh6*-Cre mice in response to HFD consumption, suggesting that HFD consumption upregulates PPARα targets in cardiomyocytes (Figure 1B).

To investigate whether PPARα directly regulates the target genes we identified, ChIP-Seq with anti-PPARα antibody was performed. PPARα was localized near the PPAR response element (PPRE) in the promoters of known PPARα target genes such as *Acox1*, *Cd36*, *Cpt1b*, *Ech1*, *Fatp1*, and *Pdk4*. Recruitment of PPARα localized near PPREs appeared to be enhanced at the *Acox1*, *Cd36*, *Cpt1b*, and *Pdk4* promoters, but not at the *Ech1* or *Fatp1* (*Slc27a1*) promoters, in response to HFD consumption (Figure 1C). These results are consistent with the notion that induction of PPARα target genes occurs with or without a detectable increase in PPARα recruitment to promoters. The latter mechanism involves a ligand-dependent switch in PPARα binding partners, from transcriptional repressors to co-activators, rather than increased promoter occupancy.

To further evaluate the role of PPARα in mediating upregulation of PPARα target genes during HFD consumption, we fed cardiac-specific *Ppara*-KO (*Ppara*-cKO) and overexpression (Tg-*Ppara*) mice either an HFD or normal diet (ND). The HFD-induced upregulation of PPARα target genes was inhibited in *Ppara*-cKO mice (Figure 1D). Suppression of PPARα target gene expression was also observed in systemic *Ppara*-KO mice (Supplemental Figure 2A). On the other hand, the expression of *Acox1*, *Cpt1b*, and *Ech1* was significantly enhanced and that of *Cd36*, *Fatp1*, and *Pdk4* exhibited a trend of enhancement in Tg-*Ppara* mice. Together, these results suggest PPARα is activated in response to HFD consumption and contributes to changes in gene expression during the development of HFD-induced diastolic heart dysfunction.

### PPARα plays an essential role in mediating diastolic dysfunction in response to HFD consumption.

**Figure 2:**
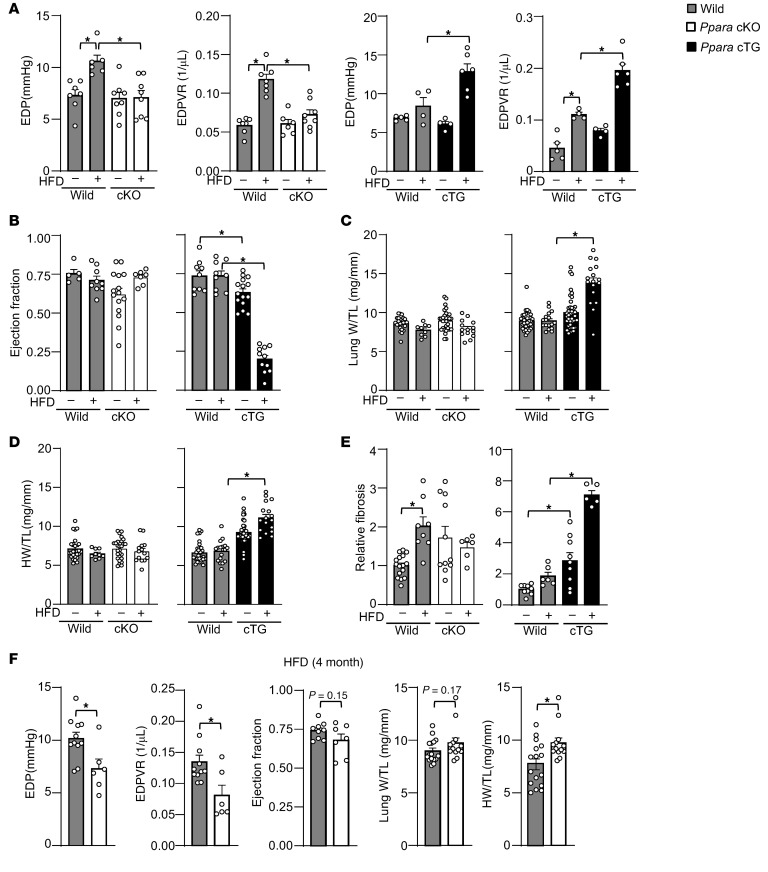
PPARα mediates HFD-induced diastolic dysfunction. (**A**) HFD-induced diastolic dysfunction is ameliorated in *Ppara-c*KO mice but exacerbated in Tg-*Ppara* mice. The HFD was provided for 1 month. (**B**) After 1 month of HFD feeding, cardiac systolic function was unchanged in *Ppara-*cKO mice but impaired in Tg-*Ppara* mice. (**C**) Ratio of lung weight (W) to tibia length (TL). (**D**) Ratio of heart weight (HW) to TL. (**E**) HFD-induced cardiac fibrosis was ameliorated in *Ppara-c*KO mice but exacerbated in Tg-*Ppara* mice. The HFD was provided for 3 months to *Ppara-*cKO mice and 1 month to Tg-*Ppara* mice. (**F**) The role of cardiomyocyte PPARα in cardiac pathology during prolonged HFD feeding. *Ppara-*cKO mice were fed an HFD for 4 months. Diastolic function (EDP, EDPVR), systolic function (ejection fraction), pulmonary congestion (lung W/TL ratio), and cardiac hypertrophy (HW/TL ratio) were assessed to evaluate the progression of cardiac pathology. The numbers of mice examined in each experimental group were as follows: 4–7 (**A**); 5–15 (**B**); 9–41 (**C**); 9–40 (**D**); 5–16 (**E**); and 6–11 (EDP and EDPVR), 7–9 (ejection fraction), and 13–16 (lung W/TL and HW/TL ratio) (**F**). Statistical significance is indicated by an asterisk and was assessed using the Kruskal-Wallis test for Tg-*Ppara* mice (**C**), Tg-*Ppara* mice (**D**), and *Ppara-*cKO mice (**E**), the Student’s *t* test (**F**) (EDP, ejection fraction), the Mann-Whitney *U* test (**F**) (EDPVR, LW/TL, and HW/TL), and ANOVA for other comparisons (**A**–**E**).

To investigate the role of PPARα in mediating HFD-induced diastolic dysfunction, *Ppara*-cKO and Tg-*Ppara* mice were fed an HFD for 1 month. Diastolic dysfunction, as evidenced by increased EDP and EDPVR, was ameliorated in *Ppara*-cKO mice but exacerbated in Tg-*Ppara* mice compared with in respective WT mice (Figure 2A). LVEF was comparable between *Ppara*-cKO and WT mice after both ND and HFD consumption (Figure 2B). Overexpression of *Ppara* alone promotes metabolic derangement, which is enhanced by HFD consumption, due to increased ligand binding to PPARα (20, 33). As reported previously (33, 34), Tg-*Ppara* mice exhibited systolic dysfunction during ND consumption, and this was exacerbated during HFD consumption (Figure 2B). *Ppara*-cKO mice did not have lung congestion, as assessed by the lung weight-to-tibia length ratio, nor did they develop cardiac hypertrophy, as evaluated by the heart weight-to-tibia length ratio (Figure 2, C and D). In contrast, Tg*-Ppara* mice had significant lung congestion and cardiac hypertrophy under HFD conditions (Figure 2, C and D), consistent with previous reports (20, 33). Cardiac fibrosis was observed after 3 months of HFD consumption in WT mice but was not significant in *Ppara*-cKO mice (Figure 2E). Because Tg-*Ppara* mice fed an HFD had a high mortality rate (data not shown), cardiac fibrosis was examined after only 1 month of HFD consumption (Figure 2E). Although cardiac fibrosis was not observed at this time point in WT mice, there was significant fibrosis in Tg-*Ppara* mice after both ND and HFD consumption. Systemic *Ppara*-KO mice had similar outcomes to those of *Ppara*-cKO mice (Supplemental Figure 2, B–F). These results suggest PPARα in cardiomyocytes plays an essential role in the development of HFD-induced diastolic dysfunction.

To test whether PPARα continues to contribute to diastolic dysfunction during prolonged HFD exposure, *Ppara-*cKO mice were fed an HFD for 4 months (Figure 2F). HFD-induced increases in EDP and EDPVR remained inhibited in *Ppara-*cKO mice, suggesting that cardiomyocyte PPARα continues to mediate diastolic dysfunction during extended metabolic stress. However, *Ppara-*cKO mice exhibited a trend toward reduced LVEF and increased lung weight, and cardiac hypertrophy was enhanced in *Ppara*-cKO mice compared with in WT control mice. These findings suggest that although *Ppara* deletion protects against diastolic abnormalities, it may impair the metabolic adaptation of the heart during sustained lipid overload.

### PPARα mediates HFD-induced IL-6 production in cardiomyocytes, thereby promoting diastolic dysfunction.

**Figure 3:**
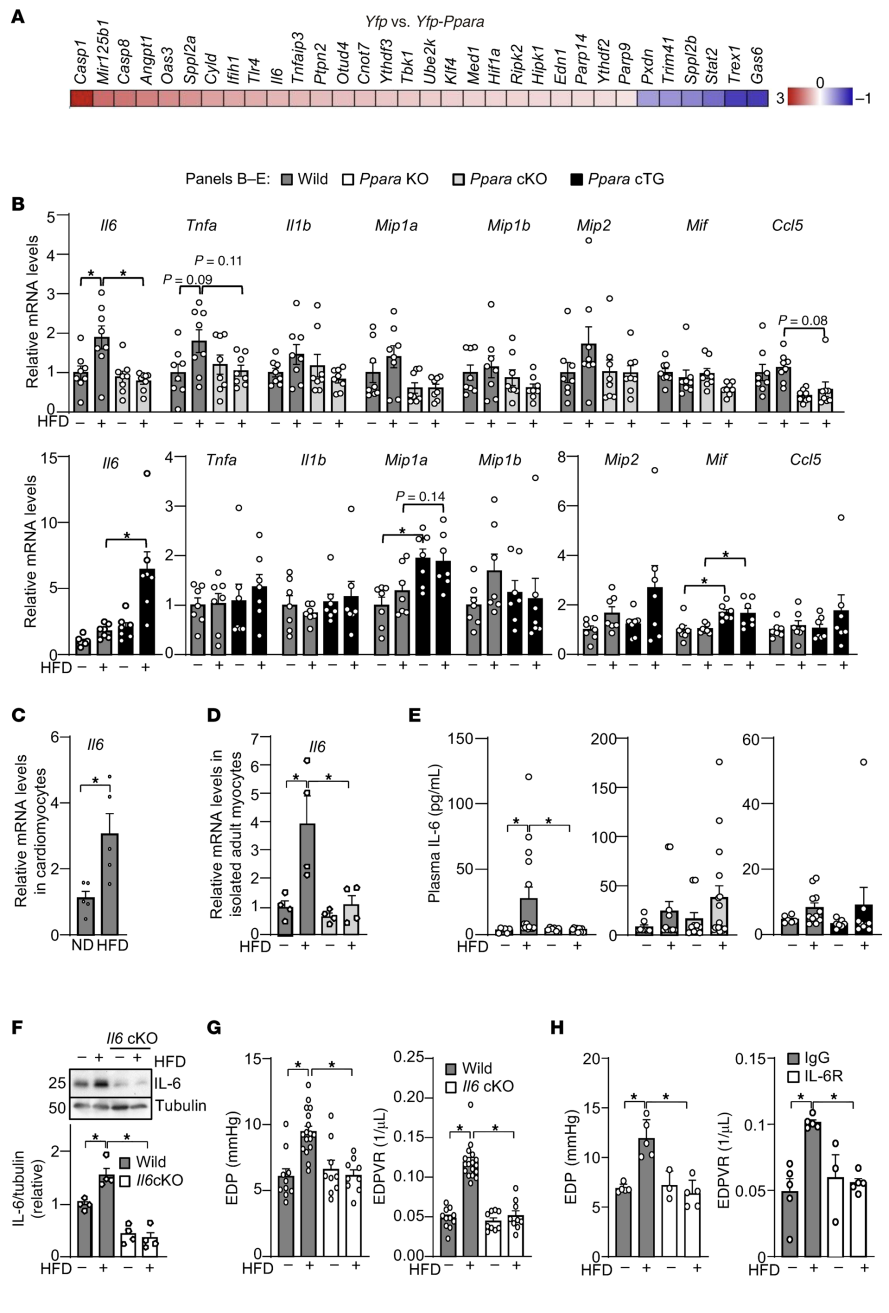
PPARα mediates HFD-induced inflammatory cytokine expression, thereby promoting diastolic dysfunction. (**A**) PPARα upregulates cytokine- and inflammation-related genes. RNA-Seq was performed in H9c2 cells with *Yfp*-*Ppara* overexpression. Among the genes listed in Gene Ontology biological process “regulation of response to cytokine stimulus,” those whose expression was significantly changed by PPARα overexpression are shown in a heatmap. (**B**) Expression levels of indicated cytokines after 1 month of HFD consumption in *Ppara-*cKO and Tg-*Ppara* mice. *n* = 6–8. (**C**) Cardiomyocytes express *Il6*. After 1 month of HFD consumption, *Il6* expression was examined in Ribo-tag mice. (**D**) PPARα-dependent *Il6* induction in adult cardiomyocytes. The mRNA level of *Il6* was examined in adult cardiomyocytes isolated from *Ppara-*cKO mice fed an HFD for 1 month. (**E**) Plasma levels of IL-6 in *Ppara-*KO, *Ppara-*cKO, and Tg-*Ppara* mice. (**F**) IL-6 induction in response to an HFD were inhibited in *Il6-*cKO mice. Protein levels of IL-6 were examined in heart lysates of *Il6-*cKO mice after 1 month of HFD consumption. (**G**) HFD-induced diastolic dysfunction was ameliorated in *Il6-*cKO mice. (**H**) HFD-induced diastolic dysfunction was ameliorated with anti–IL-6R antibody. The numbers of mice examined in each experimental group were as follows: 7–8 (**B**), 5 (**C**), 4 (**D**), 6–16 (**E**), 4(**F**), 9–17 (**G**), and 3–5 (**H**). Statistical significance is indicated by an asterisk and was assessed using the Kruskal-Wallis test for Tg-*Ppara* mice (**C**), Tg-*Ppara* mice (**D**), and *Ppara-*cKO mice (**E**), and ANOVA for other comparisons (**A**–**E**).

The low-grade inflammation induced by obesity is thought to promote diastolic dysfunction (35). Cardiomyocytes produce inflammatory cytokines, including TNF-α and IL-6 (36). To assess whether PPARα modulates immune and inflammatory gene expression, we conducted RNA-Seq of H9c2 cardiomyoblasts in the presence or absence of *Ppara* overexpression. Among the inflammatory genes categorized under the Gene Ontology biological process “regulation of response to cytokine stimulus,” more genes were upregulated than suppressed by *Ppara* overexpression, with upregulated genes including *Cas1*, *Tlr4*, *Il6*, and *Ripk2*, suggesting that PPARα activates these immune and inflammatory genes in a cell-autonomous manner (Figure 3A).

We next examined the role of PPARα in mediating HFD-induced inflammatory cytokine expression in the heart. Because diastolic dysfunction was observed within 1 month of HFD consumption, we fed mice an ND or HFD for 1 month. We examined expression of 4 major inflammatory cytokine genes (*Il1a*, *Il1b*, *Il6*, and *Tnf*a) reported to be upregulated in obesity (37). As shown in Figure 3B, cytokine expression tended to increase in response to HFD consumption, an effect that was attenuated in *Ppara*-cKO mice but enhanced in Tg-*Ppara* mice. Systemic *Ppara*-KO mice exhibited similar outcomes to those of *Ppara*-cKO mice (Supplemental Figure 2G). *Il1a* expression was not detectable in any of the mice, suggesting that *Il1α* may be silenced in the heart. HFD-induced cytokine expression in the heart could play a role in immune cell infiltration. However, FACS analyses revealed no significant changes in leukocytes, macrophages, monocytes, neutrophils, or T-cells in the heart in response to HFD consumption for 1 month compared with in response to pressure overload, which served as a positive control for our analyses (Supplemental Figure 3A). As shown in Supplemental Figure 3B, CD68, a macrophage marker, was also not significantly affected by HFD consumption in WT, *Ppara*-KO, or *Ppara*-cKO mice. However, a trend toward an increase in CD68 was observed in Tg-*Ppara* mice fed an HFD. These results suggest WT mice develop diastolic dysfunction after 1 month of HFD consumption, even before histological manifestation of inflammatory cell infiltration.

To investigate the role of macrophages in diastolic dysfunction, these cell types were depleted with clodronate (38) (Supplemental Figure 3C). Macrophage depletion did not inhibit HFD-induced diastolic dysfunction, suggesting macrophages do not play a major role in mediating diastolic dysfunction in response to HFD consumption (Supplemental Figure 3D).

Among the cytokines we tested, *Il6* was one of the most prominently upregulated during HFD consumption, and its upregulation was PPARα dependent in the heart (Figure 3, A and B). We confirmed the statistical significance in analyses of male and female mice combined and male mice alone, but not in female mice alone, due to the insufficient availability of mice (Supplemental Figure 3E). HFD-induced *Il6* upregulation in cardiomyocytes was verified with Ribo-tag: *Myh6*-Cre mice (Figure 3C). Upregulation of *Il6* mRNA in response to HFD consumption was inhibited in adult cardiomyocytes isolated from *Ppara*-cKO mice (Figure 3D). Thus, HFD induces *Il6* in cardiomyocytes in a PPARα-dependent manner. To further investigate the cellular source of IL-6 within the heart, cardiac tissue was fractionated into cardiomyocyte and noncardiomyocyte fractions. IL-6 expression was significantly induced in cardiomyocytes, whereas only a modest increase was observed in noncardiomyocytes (Supplemental Figure 3F), supporting that cardiomyocytes are the primary contributors to IL-6 production in response to HFD consumption.

To investigate whether PPARα mediates systemic IL-6 induction in response to HFD consumption, the IL-6 level in the plasma was evaluated. HFD increased the plasma level of IL-6 in WT mice, an effect that was completely inhibited in *Ppara*-KO mice (Figure 3E). However, HFD-induced increases in the plasma IL-6 level were not significantly altered in *Ppara*-cKO or Tg-*Ppara* mice (Figure 3E). Thus, PPARα in cardiomyocytes does not contribute to the increased level of plasma IL-6 in response to HFD consumption. Furthermore, diastolic dysfunction was associated with the IL-6 level in cardiomyocytes, rather than that in the plasma, in *Ppara*-cKO and Tg-*Ppara* mice.

To investigate whether IL-6 produced by cardiomyocytes contributes to diastolic dysfunction, cardiac-specific *Il6*-KO (*Il6-*cKO) mice were generated. *Il6-*cKO mice did not show any sign of cardiac hypertrophy or lung congestion at baseline (Supplemental Table 1). The IL-6 level in the heart, evaluated with immunoblot analyses, was significantly elevated in response to 1 month of HFD consumption in WT mice (Figure 3F). However, it was significantly lower in *Il6-*cKO mice both at baseline and in response to HFD consumption, suggesting cardiomyocytes are the major cell type in which IL-6 is produced at baseline and upregulated after 1 month of HFD consumption. HFD-induced diastolic dysfunction was completely prevented in *Il6-*cKO mice, suggesting IL-6 produced by cardiomyocytes plays a key role in diastolic dysfunction development (Figure 3G). Taken together, these results suggest PPARα in cardiomyocytes contributes to diastolic dysfunction through local production of IL-6 in cardiomyocytes. We also used anti–IL-6R antibody to block IL-6 signaling. Although a control antibody did not affect the development of diastolic dysfunction in response to HFD consumption, anti–IL-6R antibody fully ameliorated it (Figure 3H). Thus, IL-6 signaling appears to be a promising target for the treatment of diastolic dysfunction associated with obesity.

To investigate downstream signaling events of IL-6, we assessed IL-6Ra levels and phosphorylation of Stat3 at Y705 in the heart (39). Among the downstream mediators of IL-6 signaling, Stat3 is a key effector that not only drives transcription but also interacts cooperatively with other inflammatory regulators, including NF-κB, AP-1, and HIF-1α, thereby amplifying inflammatory signaling cascades (40). Although IL-6Ra expression was not significantly changed, Stat3 phosphorylation was significantly increased in WT mice after HFD consumption and was attenuated in *Ppara*-cKO but enhanced in Tg-*Ppara* mice (Supplemental Figure 3, G and H). These results suggest HFD activates Stat3 in a PPARα-dependent manner, independent of changes in IL-6Ra expression.

High IL-6 levels suppress PPARα expression in hepatocytes through Stat1/3 signaling (41). Although IL-6–dependent Stat3 phosphorylation was evident in HFD-fed mice, cardiac PPARα expression was unchanged in either HFD-fed or *Il6-*cKO mice (Supplemental Figure 3I). These results suggest IL-6 promotes diastolic dysfunction in the heart through activation of Stat3 signaling, rather than through suppression of PPARα.

Gain of PPARα function promotes lipotoxicity, characterized by increased myocardial triglyceride content, and contributes to cardiac dysfunction (33). To assess the role of endogenous PPARα in this process, we measured myocardial triglyceride levels in *Ppara-*KO mice. The HFD-induced increase in triglyceride content was significantly attenuated in *Ppara-*KO mice, indicating that lipotoxicity under HFD conditions is PPARα dependent (Supplemental Figure 3J). To determine whether IL-6 mediates this lipotoxic response, we measured myocardial triglyceride content in *Il6-*cKO mice. In contrast to *Ppara-*KO mice, *Il6-*cKO mice did not have significant differences in triglyceride accumulation after HFD feeding (Supplemental Figure 3K), which suggests IL-6 does not contribute to lipid accumulation. Taken together, these results indicate IL-6 mediates only a subset of PPARα-dependent cardiac pathologies, such as diastolic dysfunction, but not lipotoxicity.

### Verification of Il6 induction in cardiomyocytes via single-cell RNA-Seq.

To further verify that a HFD induces PPARα target gene expression and *Il6* upregulation in cardiomyocytes, single-cell RNA-Seq (scRNA-Seq) was performed using mouse heart tissues. In this experiment, cardiomyocytes constituted the majority of sequenced cells. Compared with mice fed an ND, only a limited population of cardiomyocytes was recovered from HFD-fed mice (Supplemental Figure 4A). This reduction likely indicates increased fragility of cardiomyocytes in mice consuming an HFD, resulting in lower recovery rates during the cell-labeling process. This may compromise the analysis of various cardiomyocyte clusters in the context of HFD consumption. In addition, during barcoding and library preparation, cells with elevated mitochondrial RNA content, a marker of cellular stress (42), were excluded during quality-control filtering.

Despite this limitation, the analysis of cardiomyocyte clusters confirmed upregulation of PPARα target genes, including *Acox1*, *Cpt1b*, *Cd36*, *Ech1*, and *Acadm*, in cardiomyocytes after HFD consumption (Supplemental Figure 4B). Consistent with the presence of low-grade inflammation associated with obesity, a modest increase in *Il6* expression was also observed in cardiomyocytes from HFD-fed mice (Supplemental Figure 4C). In contrast, the expression patterns of *Il1b*, *Tnfα*, and *Mif* differed between qPCR of the whole heart (Figure 3B) and scRNA-Seq of cardiomyocyte clusters (Supplemental Figure 4C), possibly due to differences in the specific cell populations analyzed by each method. A Kyoto Encyclopedia of Genes and Genomes pathway analysis revealed that HFD consumption stimulated several inflammatory signaling pathways, including primary immunodeficiency, T cell receptor signaling, and NF-κB signaling (Supplemental Figure 4D). Bioinformatics analyses also showed activation of the JAK-STAT pathway, a downstream signaling pathway activated by IL-6. Collectively, these data confirmed activation of an inflammatory signature and enhanced PPARα and IL-6 signaling in cardiomyocytes in response to HFD consumption.

### PPARα mediates fatty acid–induced Il6 production.

**Figure 4:**
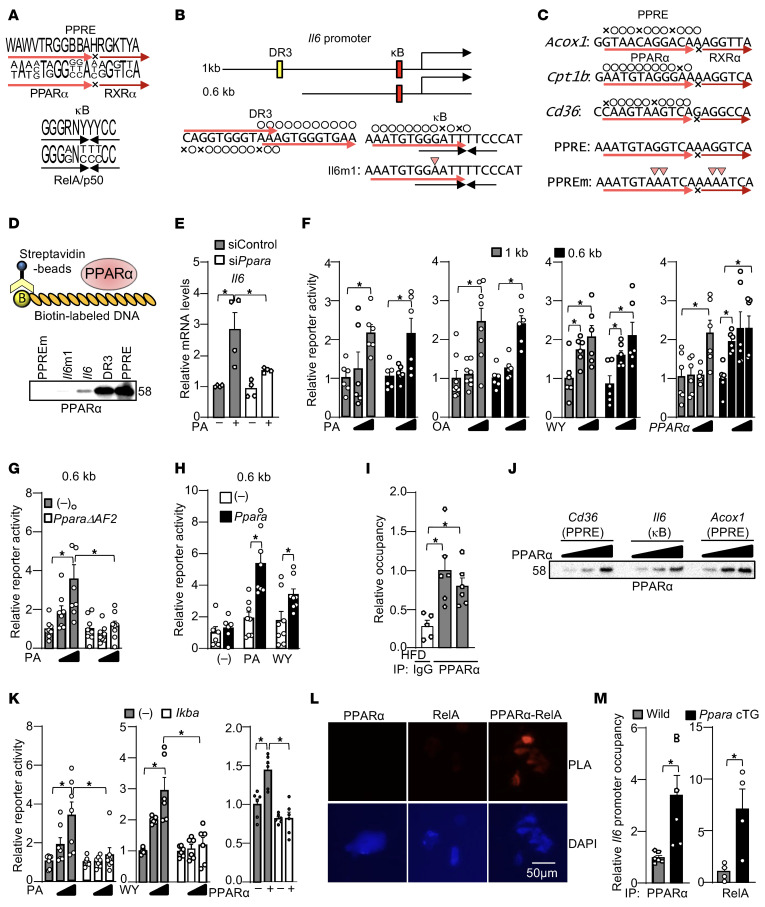
PPARα induces *Il6*. (**A**) Consensus sequences of PPRE and NF-κB binding element. (**B**) Schematic representation of mouse *Il6* promoter. (**C**) The PPRE DNA sequences used in this study. (**D**) PPARα binds to the DR3 and κB elements in the *Il6* promoter in vitro. Data shown are from a single experiment. However, key binding features, including stronger binding to the consensus PPRE than to the *Il6* κB element or its mutant, were reproducibly observed in at least 2 independent experiments (e.g., Figure 5G) (**E**) PPARα mediates the *Il6* induction response to PA. *n* = 4. The immunoblot shown is representative of 3 independent experiments. (**F**) PPARα activators activate *Il6* transcription through the 1 kb and 0.6 kb *Il6* promoters. *n* = 6–8. (**G**) Fatty acid–induced *Il6* promoter activation is inhibited by dominant negative PPARα (PPARαΔAF2). *n* = 8. (**H**) PPARα mediates PA- and WY-induced *Il6* promoter activation. *n* = 6–8. (**I**) PPARα is recruited to the *Il6* promoter. *n* = 5–6. (**J**) PPARα has an equivalent affinity for the κB element in the *Il6* promoter and PPREs in the *Cd36* and *Acox1* promoters. Data are representative of 2 independent experiments. (**K**) PA-, WY-, and PPARα-induced *Il6* promoter activation is inhibited by a super suppressor form of IκBα (IκBαM). *n* = 6–7. (**L**) Proximity ligation assay (PLA) assay shows the binding of PPARα to NF-κB. (**M**) PPARα and RelA bind to the *Il6* promoter. *n* = 4–6. Statistical significance was assessed using the Kruskal-Wallis test for the 1 kb promoter stimulated with OA and the 0.6 kb promoter stimulated by *Ppara* overexpression (**F**), and for PA stimulation (**K**); the Mann-Whitney *U* test for PA stimulation (**H**); Student’s *t* test (**M**); and ANOVA for all other comparisons.

To gain insight into how PPARα induces *Il6* in response to HFD consumption, we inspected PPARα binding sequences in the mouse *Il6* promoter. Although the authentic PPRE/DR1 type of PPARα/RXR heterodimer binding sequence was not found, 3 potential monomeric PPARα binding sequences were identified in the *Il6* promoter (Figure 4, A and). Two of them were aligned with a 3-nucleotide spacer, designated as direct repeat 3 (DR3). The third PPARα binding sequence overlapped with a half site of the NF-κB binding sequence to which a monomeric NF-κB subunit, such as RelA or p50, can bind; this is designated as the κB element. To evaluate PPARα binding to the DR3 and the κB element, in vitro DNA binding assays were performed with recombinant PPARα and biotin-labeled DNA comprising WT and mutated versions of the κB element and PPRE, as shown in Figure 4, B and C. The purity of the recombinant proteins used in this study, including PPARα, RXRα, RelA, and p50, is shown in Supplemental Figure 5A. The DNA binding assay showed that PPARα binds to both the DR3 and the κB element (Figure 4D). PPARα binding to the κB (*Il6* κB) element was weaker than to the DR3 but was further reduced when a single-nucleotide mutation (*Il6*m1) was introduced into the κB element, indicating sequence-dependent interaction (Figure 4D).

To test whether PPARα mediates fatty acid–induced *Il6* upregulation in cultured cardiomyocytes, PPARα was knocked down with siRNA. Palmitic acid (PA), a fatty acid, upregulated *Il6* mRNA, an effect that was inhibited by PPARα knockdown (Figure 4E and Supplemental Figure 5B). Thus, PPARα mediates fatty acid–induced *Il6* transcription in cardiomyocytes.

Because PPARα strongly binds to the DR3, we originally hypothesized that the DR3 mediates PPARα-mediated *Il6* promoter activation. However, both fatty acid ligands, such as PA, oleic acid (OA), and an artificial ligand (WY14643 [WY]), and overexpression of PPARα activated transcription of a reporter gene harboring the 0.6 κb *Il6* promoter without the DR3 (Figure 4F). Thus, the DR3 is not necessary for PPARα-induced *Il6* promoter activation. To test whether PPARα mediates fatty acid–induced activation of the *Il6* promoter, the effect of PPARαΔAF2, a dominant negative mutant of PPARα (43), was examined. PA- and OA-induced *Il6* promoter activation was inhibited by PparaΔAF2 (Figure 4G). We also exogenously expressed *Ppara* in Cos7 cells to further examine the role of PPARα in *Il6* induction. PA and WY significantly activated the *Il6* promoter in the presence of exogenous PPARα (Figure 4H).

To test whether PPARα binds to the *Il6* promoter in the heart in vivo, chromatin immunoprecipitation assays were performed. PPARα bound to a flanking region of the κB element on the *Il6* promoter under both ND and HFD consumption conditions (Figure 4I). To test whether PPARα binds to the κB element with an affinity equivalent to that for endogenous PPREs, the effect of PPARα dose upon binding to the κB element was evaluated via pull-down assays with biotin-labeled oligonucleotides harboring endogenous PPRE sequences derived from the *Cd36* and *Acox1* promoters. The binding affinity of PPARα for the κB element was slightly stronger than for the PPRE derived from the *Cd36* promoter and weaker than for the PPRE derived from the *Acox1* promoter, suggesting the binding affinity of PPARα for the κB element is comparable to that of endogenous PPREs (Figure 4J). These results suggest PPARα mediates fatty acid–induced *Il6* promoter activation most likely through direct interaction with the κB element.

### NF-κB mediates fatty acid–induced Il6 production.

Because PPARα binds to the κB element in vitro, we next investigated the role of NF-κB in mediating PPARα-induced *Il6* promoter activation. To this end, we evaluated the effect of a super-suppressor form of IκBα (IκBαM), which inhibits NF-κB. IBαM inhibited PA, WY, and *Ppara* overexpression-induced *Il6* promoter activation, which suggests NF-κB is required for PPARα-induced *Il6* induction (Figure 4K). In Tg-*Ppara* mice expressing Flag-tagged PPARα, both RelA and p50 of NF-κB were co-immunoprecipitated with Flag-PPARα, a finding suggesting that PPARα binds to NF-κB in the heart. The level of interaction between PPARα and RelA/p50 was not altered in HFD consumption (Supplemental Figure 5C). Proximity ligation assays revealed interaction between PPARα and the RelA NF-κB subunit in DAPI-positive nuclei in cultured cardiomyocytes (Figure 4L). Recruitment of PPARα and RelA to the *Il6* promoter proximal to the κB element was enhanced in Tg-*Ppara* mice, suggesting PPARα can recruit NF-κB (Figure 4M). These results suggest NF-κB plays an essential role in PPARα-induced *Il6* transcription, possibly through interaction with PPARα.

### PPARα heterodimerizes with NF-κB and binds to the κB element.

The PPARα binding sequence in the 0.6 kb region of the *Il6* promoter partially overlaps with the κB element (44). The 5′ half of the κB element in the *Il6* promoter possesses a PPARα-preferred binding sequence, whereas the 3′ half of the κB element represents a typical binding sequence for monomeric Rel or p50 proteins (YYCC; Y: T or C) rather than RXRs (RGKTYA; G:A or G, K: G or T) (Figure 4A). Because direct binding of PPARα to RelA has been reported (45), we hypothesized that PPARα heterodimerizes with either RelA or p50 and binds to the κB element.

**Figure 5:**
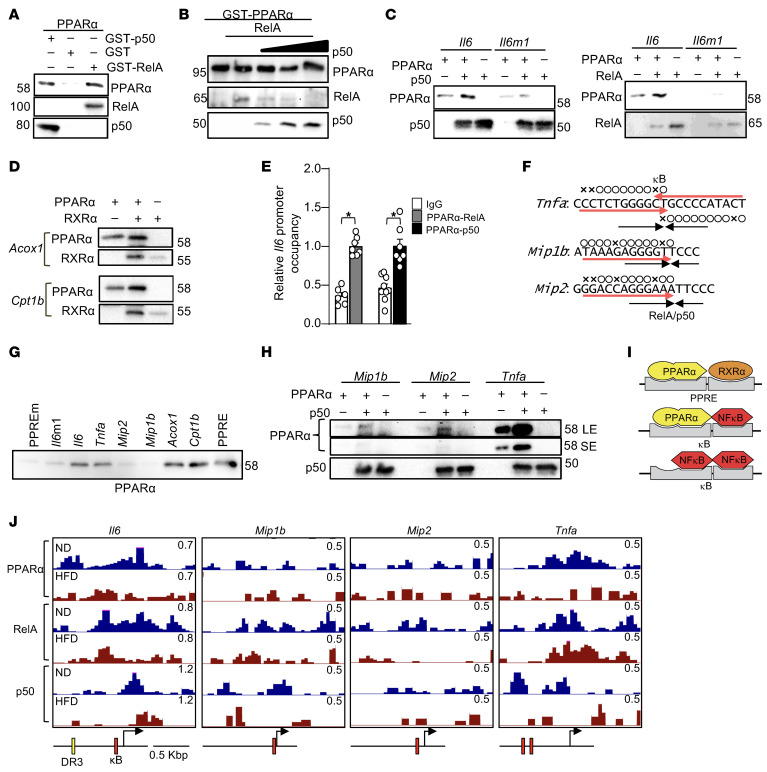
PPARα forms a heterodimer with NF-κB. (**A**) PPARα directly binds to RelA and p50 in vitro. Data shown are from a single experiment; the interaction was independently examined using a reciprocal assay in **B**. (**B**) PPARα binds to NF-κB proteins as a heterodimer entity. (**C**) The binding of PPARα to the κB element is enhanced by NF-κB proteins. Each panel represents an independent immunoblot derived from a separate membrane. (**D**) The binding of PPARα to the PPRE is enhanced by RXRα. Data shown are from a single experiment. (**E**) PPARα and NF-κB colocalize to the *Il6* promoter. Double ChIP assays were performed with the indicated antibodies and mouse hearts. *n* = 6–9. (**F**) The endogenous κB sequences in *Tnfa, Mip1b,* and *Mip2* promoters. (**G**) PPARα binds to a subset of, but not all, endogenous κB elements. (**H**) The binding of PPARα to the κB element is enhanced by NF-κB proteins. Data shown are from a single experiment; similar differential binding of PPARα to these elements was independently reproduced in (**G**). (**I**) Schematic representation of PPARα binding to PPRE and κB element. (**J**) ChIP-Seq shows that PPARα binds to the *Il6* and *Tnfa* promoters. The *y* axis in the University of California, Santa Cruz, genome browser is indicated at top right (0.5–1.2) of each chart. ChIP-Seq was performed once using pooled heart samples from 3 mice after 1 month of HFD feeding. Data in **B**, **C**, and **G** are representative of 2 independent experiments. Statistical significance (*P* < 0.05) is indicated by an asterisk and was assessed using Student’s *t* test (**E**).

To test this hypothesis, in vitro protein-protein and protein-DNA binding assays were performed using recombinant proteins and biotin-labeled DNAs. In vitro binding assays showed that PPARα directly binds to p50 as well as to RelA (Figure 5A). In vitro competition assays showed the binding of PPARα to RelA was competitively inhibited by p50, suggesting that both RelA and p50 bind to PPARα in a competitive manner (Figure 5B) such that PPARα can heterodimerize with either RelA or p50. To investigate whether the binding of PPARα to the κB element is promoted by NF-κB proteins, DNA binding assays were performed. Binding of PPARα to the κB element was promoted in the presence of either p50 or RelA (Figure 5C). In contrast, binding of p50 or RelA to the κB element was partially reduced by PPARα, but this was not observed when the binding of PPARα to the κB element was inhibited by the introduction of a mutation in the κB element (*Il6*m1) (Figure 5C). This suggests the reduced binding of p50 and RelA to the κB element in the presence of PPARα is likely due to conversion from p50/p50 or RelA/RelA homodimers to PPARα/p50 or PPARα/RelA heterodimers. Thus, the binding of PPARα to the κB element is promoted by heterodimerization with NF-κB proteins, just as binding of PPARα to the PPRE is promoted by heterodimerization with RXR. Through the DNA binding assays, we verified that the binding of PPARα to the endogenous PPREs derived from the *Acox1* and *Cpt1b* promoters was enhanced by RXRα (Figure 5D). Double ChIP assays revealed that PPARα and NF-κB were colocalized at the *Il6* promoter (Figure 5E). These results suggest PPARα heterodimerizes with NF-κB, enhancing binding to the κB element.

To test whether PPARα binds to other κB elements besides the one in the *Il6* promoter, in vitro DNA binding assays were conducted with biotin-labeled DNA comprising κB elements from the *Tnfa*, *Mip1b*, and *Mip2* promoters (Figure 5, F and G). PPARα strongly bound to the κB elements derived from the *Il6* and *Tnfa* promoters but not to those from the *Mip2* or *Mip1b* promoters (Figure 5G). To investigate whether the binding of PPARα to these κB elements is enhanced by NF-κB, in vitro DNA binding assays were performed. Binding of PPARα to the κB element derived from the *Tnfα* promoter was significantly enhanced by p50 (Figure 5H). Although the binding of PPARα to the κB elements derived from the *Mip2* and *Mip1b* promoters was also enhanced by p50, there was still significantly less PPARα binding to these κB elements than to that from the *Tnfa* promoter. These results suggest PPARα binds to only a subset of κB elements, and this binding is enhanced by its heterodimerization with NF-κB proteins. This subset of κB elements appears to possess a preferred PPARα-binding sequence in its half site, where either authentic NF-κB dimer or PPARα/NF-κB heterodimer can bind (Figure 5I).

To evaluate PPARα and NF-κB binding to the promoters of inflammatory genes in the heart, ChIP-Seq was performed with anti-PPARα, anti-RelA, and anti-p50 antibodies. The peaks of PPARα binding were observed in the proximity of the DR3 and κB elements in the *Il6* promoter under ND and HFD feeding conditions (Figure 5J). RelA and p50 were also localized in the proximity of the κB element, but their occupancy declined under HFD feeding conditions. These data suggest HFD-induced *Il6* expression is not due to increased PPARα–NF-κB complex formation but rather to a shift in the transcriptional activity of a preexisting complex caused by ligand binding. We propose that fatty acid ligands activate PPARα within the complex, leading to coactivator recruitment and transcriptional activation, analogous to classical PPARα ligand responses (16, 46), rather than inducing new heterodimerization with NF-κB in response to HFD.

As in the oligonucleotide in vitro DNA binding assays, PPARα bound to the *Tnfα* promoter but not significantly to the *Mip1b* and *Mip2* promoters (Figure 5J). These results suggest PPARα transcribes a subset of NF-κB target genes via direct binding to their promoters.

### PPARα/NF-κB heterodimerization is required for fatty acid–induced Il6 promoter activation.

**Figure 6:**
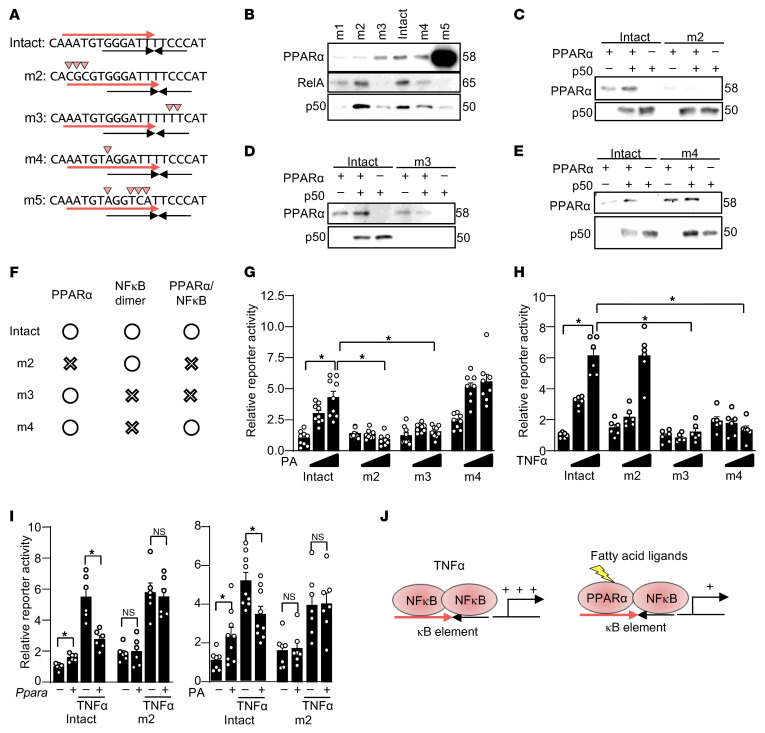
PPARα/NF-κB heterodimer is required for fatty acid–induced *Il6* promoter activation. (**A**) The κB element mutations used in this study. (**B**–**E**) In vitro DNA binding assays with the indicated recombinant proteins and mutated κB elements. Data in (**B**) are from a single experiment; the relative binding patterns observed in (**B**) were independently reproduced in (**C**–**E**), which are representative of at least 2 independent experiments. (**F**) Summary of PPARα and NF-κB binding to the κB mutants. (**G**) PPARα/NF-κB heterodimer is required for PA-induced *Il6* promoter activation. *n* = 8–9. (**H**) PPARα is not required for TNF-α–induced *Il6* promoter activation. *n* = 6. (**I**) DNA binding of PPARα is required for fatty acid–induced *Il6* promoter activation and for inhibition of TNF-α–induced *Il6* promoter activation. *n* = 6–9. (**J**) Schematic representation of how TNF-α–induced *Il6* promoter activation is mediated by NF-κB dimer, whereas fatty acid–induced activation is mediated by PPARα/NF-κB heterodimer. Statistical significance (*P* < 0.05) is indicated by an asterisk and was assessed using ANOVA (**G** and **H**) and Student’s *t* test (**I**).

To verify that the binding of PPARα to the κB element is enhanced by heterodimerization with NF-κB proteins, we performed in vitro DNA binding assays using oligonucleotides harboring the *Il6* promoter with mutated κB elements (namely, m2, m3, m4, and m5) and with recombinant proteins, including PPARα, RelA, and p50 (Figure 6A). In m5, we mutated the κB element in a way that allows PPARα to bind. This mutant was used as a positive control for PPARα binding and as a negative control for NF-κB binding (Figure 6B). In m2, we mutated the PPARα binding region located outside of the NF-κB binding region. This mutant can bind an NF-κB, but not PPARα, dimer (Figure 6B). Although binding of p50 to the WT κB element was reduced in the presence of PPARα, binding of p50 to m2 was not affected, suggesting that binding of PPARα to the half site of the κB element reduces p50 binding, most likely due to the conversion from p50/p50 homodimers to PPARα/p50 heterodimers (Figure 6C). In m3, we mutated the 3′ half site of the κB element to inhibit binding of NF-κB without affecting the binding of monomeric PPARα. Although binding of PPARα to the WT κB element was enhanced by p50, binding of PPARα to m3 was not enhanced in the presence of p50 (Figure 6D). These results suggest the binding of PPARα to the κB element is enhanced when NF-κB binds to the 3′ half site of the κB element. In m4, to inhibit binding of an NF-κB dimer but allow binding of a PPARα/NF-κB heterodimer, we introduced a point mutation in the 5′ half site, which partially reduces the DNA binding of NF-κB without affecting PPARα binding (Figure 6B). Although binding of p50 to m4 was reduced in the absence of PPARα, it was not reduced in the presence of PPARα (Figure 6E). Rather, the binding of p50 to m4 was promoted in the presence of PPARα. This suggests the presence of PPARα supports binding of p50, most likely through formation of the PPARα/p50 heterodimer. Taken together, these results suggest PPARα heterodimerizes with NF-κB and binds to the κB element.

Based upon the results of the experiments shown in Figure 6, A–E, how monomeric PPARα, NF-κB dimer, and PPARα/NF-κB heterodimer bind to these mutants is summarized in Figure 6F. To investigate whether PPARα and NF-κB heterodimerization is required for *Il6* promoter activation, reporter gene assays were performed using luciferase reporter genes harboring the 1 kb *Il6* promoter with mutations corresponding to either m2, m3, or m4. PA-induced *Il6* promoter activation was abolished when PPARα/NF-κB heterodimer binding was inhibited in reporters harboring m2 or m3. In contrast, PA-induced *Il6* promoter activation was not inhibited when NF-κB dimer binding was inhibited but PPARα/NF-κB heterodimer binding was allowed in the reporter harboring m4 (Figure 6G). These results suggest PPARα/NF-κB heterodimer, rather than authentic NF-κB dimer, is required for fatty acid–induced *Il6* promoter activation. In contrast, TNF-α–induced *Il6*-promoter activation was inhibited when NF-κB dimer binding was inhibited in reporters harboring m3 or m4, whereas it was not inhibited when PPARα/NF-κB heterodimer binding was inhibited but NF-κB dimer binding was allowed in the reporter harboring m2 (Figure 6H). These results suggest that authentic NF-κB dimer, rather than PPARα/NF-κB heterodimer, is required for TNF-α–induced *Il6* promoter activation. In summary, PPARα/NF-κB heterodimer is required for fatty acid–induced *Il6* induction through the κB element, whereas PPARα is not essential for *Il6* induction by other stimuli such as TNF-α.

Although our results suggest PPARα cooperatively works with NF-κB to activate transcription, previous studies have shown that PPARα inhibits NF-κB–mediated transcription in cultured cells (45, 47). We hypothesized that, compared with NF-κB dimer, PPARα/NF-κB heterodimer is less potent and, thus, NF-κB dimer-induced transcription is inhibited when more PPARα/NF-κB heterodimer is formed. To test this hypothesis, reporter gene assays were performed using the reporter harboring the m2 mutation, which allows binding of an NF-κB dimer but not a PPARα/NF-κB heterodimer. Although PPARα induced modest activation of the reporter harboring the intact *Il6* promoter, PPARα inhibited TNF-α–induced activation of this reporter (Figure 6I). In contrast, neither PPARα-induced activation nor inhibition was observed in the reporter harboring m2. The identical result was observed when we used PA instead of PPARα. Thus, although PPARα mediates fatty acid–induced *Il6* promoter activation, PPARα inhibits NF-κB activation by other stimuli such as TNF-α (Figure 6J).

### PPARα/NF-κB heterodimer contributes to obesity-induced diastolic dysfunction.

**Figure 7:**
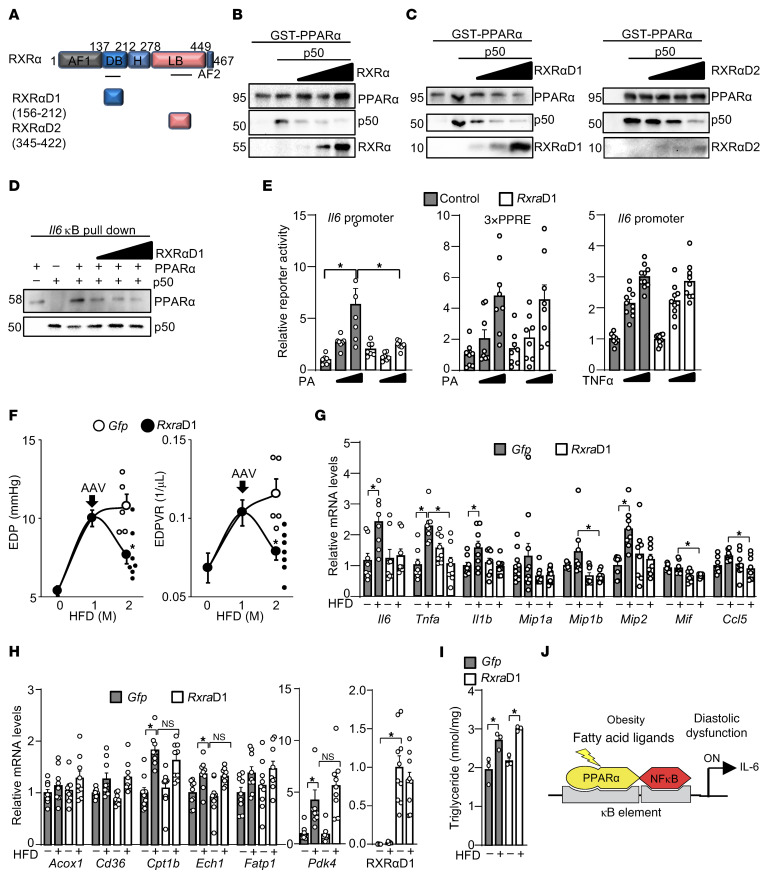
PPARα/NF-κB heterodimer mediates HFD-induced diastolic dysfunction. (**A**) Schematic representation of PPARα binding regions in RXRα. (**B**) Full-length RXRα competitively inhibits the binding between PPARα and p50. (**C**) The DNA and ligand-binding domains of RXRα competitively inhibit the binding between PPARα and p50. (**D**) RXRαD1 inhibits the binding of PPARα to the κB element but allows the binding of p50. Data in (**B**) and (**C**) were independently reproduced in 3 separate experiments, and data in (**D**) were independently reproduced in 2 separate experiments. (**E**) RXRαD1 specifically inhibits fatty acid–induced *Il6* promoter activation but not fatty acid–induced PPRE activation or TNF-α–induced *Il6* promoter activation. *n* = 7–10. (**F**) AAV-RXRαD1 ameliorates HFD-induced diastolic dysfunction. *n* = 3–5. (**G**) AAV-RXRαD1 inhibits HFD-induced cytokine expression. *n* = 8–10. (**H**) AAV-RXRαD1 does not affect authentic PPARα target gene expression. *n* = 9. (**I**) AAV-RXRαD1 does not significantly affect triglyceride content in the heart. *n* = 3. (**J**) Model of how PPARα contributes to diastolic dysfunction. Statistical significance (*P* < 0.05) is indicated by an asterisk and was assessed using Student’s *t* test (**F**) and the Kruskal-Wallis test for *Il6*, *Tnfa*, and *Mip1b* (**G**) and for *Cd36*, *Pdk4*, and *Rxra*D1 (**H**). ANOVA was used for all other comparisons.

Because the PPARα/NF-κB heterodimer promotes IL-6 production, it may contribute to diastolic dysfunction in response to HFD consumption. Because RXR is an authentic PPARα heterodimerization partner and strongly binds to PPARα (48), a short stretch of amino acids in RXR involved in the interaction with PPARα may competitively inhibit the binding of NF-κB proteins to PPARα. RXR binds to PPARα through 2 independent regions: the DNA binding domain and the ligand binding domain, designated as RXRαD1 (dimerization domain 1) and RXRαD2, respectively (Figure 7A). First, we tested whether full-length RXR inhibits the binding of PPARα to p50. In vitro binding assays revealed that full-length RXRα competitively inhibits the binding of PPARα to p50, suggesting that NF-κB and RXR bind competitively to PPARα (Figure 7B). We next tested whether RXRαD1 or RXRαD2 inhibits the binding of PPARα to NF-κB. In vitro binding assays revealed that both RXRαD1 and RXRαD2 competitively inhibit the binding of PPARα to p50 (Figure 7C). In contrast, as we have reported previously (49), RXRαD1 did not significantly inhibit the binding of PPARα to full-length RXRα (Supplemental Figure 6). These results suggest PPARα uses overlapping regions to bind to NF-κB and RXRα.

To achieve specific inhibition of PPARα/NF-κB heterodimerization, we chose RXRαD1 for two reasons. First, RXRαD1 does not inhibit the binding of PPARα to full-length RXR. Second, RXRαD2 may also competitively inhibit ligand binding to endogenous RXR. To investigate whether and how RXRαD1 inhibits the DNA binding of PPARα and NF-κB, DNA binding assays were performed. As shown in Figure 7D, the binding of PPARα to the κB element was enhanced in the presence of p50, which was inhibited by RXRαD1. In contrast, the binding of p50 to the κB element was partly inhibited by PPARα, which was normalized by RXRαD1. These results suggest RXRαD1 inhibits the binding of PPARα to the κB element, which promotes p50 homodimerization on the κB element.

To test whether RXRαD1 specifically inhibits fatty acid–induced *Il6* promoter activation, reporter gene assays were performed. PA-induced *Il6* promoter activation was inhibited by *RXRα*D1, whereas neither PA-induced PPRE activation nor TNF-α–induced *Il6* promoter activation was significantly inhibited (Figure 7E). Thus, RXRαD1 is an intriguing tool that specifically inhibits PPARα/NF-κB heterodimerization on the κB element but not NF-κB homodimerization or PPARα/RXR heterodimerization on the PPRE. To investigate the role of the PPARα/NF-κB heterodimer in mediating HFD-induced diastolic dysfunction, we generated an adeno-associated virus (AAV) to induce RXRαD1 expression (AAV-RXRαD1). HFD-induced diastolic dysfunction was partially normalized by AAV-RXRαD1 (Figure 7F). HFD-induced cytokine expression, such as *Il6*, was inhibited by AAV-RXRαD1, whereas authentic PPARα target genes involved in fatty acid metabolism were not significantly changed (Figure 7, G and H). The expression of RXRαD1 was verified (Figure 7H). To examine whether the PPARα–NF-κB interaction contributes to cardiac dysfunction by promoting lipotoxicity, we measured myocardial triglyceride content in mice transduced with AAV-RXRαD1 or AAV-GFP. AAV-RXRαD1 did not significantly alter triglyceride levels in the heart (Figure 7I). These findings suggest PPARα–NF-κB interaction does not contributes to lipotoxicity. Taken together, these results suggest the PPARα/NF-κB heterodimer mediates HFD-induced diastolic dysfunction, partly through IL-6 production.

## Discussion

It is widely accepted that inflammation and pro-inflammatory cytokines drive diastolic dysfunction and cardiomyopathy in humans and mice with obesity and diabetes (50). However, the cellular sources of cytokines and the molecular mechanisms through which cytokines are produced in the setting of obesity are poorly understood, particularly during its early stage. We here show that PPARα in cardiomyocytes is activated by HFD consumption in mice before histological and biochemical signs of inflammation are observed. PPARα in cardiomyocytes plays an essential role in mediating autocrine production of IL-6, through direct interaction with NF-κB, which, in turn, promotes diastolic dysfunction (Figure 7J).

### Pathological role of cardiomyocytes producing IL-6.

Elevated plasma levels of IL-6 are associated with an increased risk of heart failure with preserved ejection fraction (51). In addition, IL-6 infusion induces diastolic dysfunction in rats (52). Despite its importance in the pathogenesis of obesity cardiomyopathy, it has been unclear where in the body the IL-6 is produced that drives diastolic dysfunction. We showed that HFD consumption stimulates upregulation of *Il6* mRNA in cardiomyocytes. scRNA-Seq further indicated that activation of immune and inflammatory signaling pathways in cardiomyocytes takes place in obesity. HFD consumption upregulated IL-6 protein in the mouse heart, an effect that was abolished in *Il6-*cKO mice, suggesting IL-6 in the cardiac tissue is produced primarily in cardiomyocytes during HFD consumption. Furthermore, HFD-induced diastolic dysfunction was abolished in *Il6-*cKO mice. These results point to the importance of local autocrine production of IL-6 in cardiomyocytes in mediating diastolic dysfunction. Upregulation of IL-6 and diastolic dysfunction in response to HFD consumption took place before obvious histological signs of cardiac hypertrophy and fibrosis were observed (53, 54). This also supports the mechanistic role of IL-6 as an initial driver in diastolic dysfunction.

The plasma level of IL-6 was elevated in response to HFD consumption, an effect that was abolished in systemic *Ppara-*KO mice but not in *Ppara* cKO mice, suggesting PPARα in noncardiomyocytes is involved in the upregulation of IL-6 in the plasma. Because the plasma IL-6 level was not further increased in Tg-*Ppara* mice after HFD consumption compared with in control mice, we speculate that PPARα-mediated production of IL-6 in cardiomyocytes does not contribute to the increased plasma IL-6 level during HFD consumption. The identity of the cell type responsible for the HFD- and PPARα-dependent production of IL-6 in the plasma remains to be elucidated. Importantly, however, the suppression of diastolic dysfunction in *Ppara-*cKO mice was observed even when the plasma level of IL-6 remained elevated, suggesting that IL-6 produced in cardiomyocytes, rather than plasma IL-6, mediates diastolic dysfunction.

The function of PPARα as a positive mediator of IL-6 production in the context of HFD consumption shown in this study is opposite to the known function of PPARα in the literature, where PPARα negatively affects inflammatory cytokine production (45). As we will discuss, we discovered that PPARα can interact with the κB element in the promoters of selected proinflammatory genes, thereby positively regulating their expression.

### PPARα promotes IL-6 production in cardiomyocytes.

Obesity induces pathogen-independent low-grade inflammation (27). This could be a mechanism responsible for insulin resistance and diabetes (55). Despite its pathological significance, the underlying mechanism through which obesity induces inflammation is poorly understood. Protein kinase R, a serine-threonine kinase involved in protection against viral infection and endoplasmic reticular stress, plays an essential role in obesity-induced inflammation, although how protein kinase R senses obesity remains unknown (56). TLR4 may be activated by fatty acids and, in turn, activate NF-κB (57). However, it remains unclear whether fatty acids act as a direct ligand for TLR4 (58). In the present study, activation of PPARα, increased IL-6 in the heart, and the development of diastolic dysfunction were all observed in the very early phase of HFD consumption, before the infiltration of inflammatory cells was observed in the heart. This, together with the fact that HFD-induced upregulation of IL-6 in the heart was abolished in *Ppara-*cKO mice, demonstrate that myocardial upregulation of IL-6 occurs through PPARα in cardiomyocytes, independently of infiltrating pro-inflammatory cells such as macrophages. Elimination of macrophages with clodronate failed to prevent HFD-induced diastolic dysfunction. Thus, we here propose that PPARα in cardiomyocytes senses increased plasma fatty acid levels, thereby stimulating transcription of proinflammatory genes.

### The PPARα/NF-κB heterodimer promotes Il6 transcription through the κB element.

Previous studies have shown that PPARα inhibits NF-κB–induced IL-6 induction in Cos-1 cells (45). Unexpectedly, our results suggest PPARα promotes IL-6 production during HFD consumption through an NF-κB–dependent mechanism. Multiple lines of evidence support this unexpected action of PPARα. First, PPARα can stimulate the *Il6* promoter, which requires the presence of the PPARα activation domain and is stimulated in the presence of PPARα ligands. PPARα’s ability to stimulate the *Il6* reporter promoter does not require the PPRE-like element (DR3) but critically depends on the presence of both NF-κB and the κB element. Second, PPARα and p50/RelA directly interact with one another in the nucleus. Third, PPARα can interact with the κB element in the *Il6* promoter both in vitro and in vivo. DNA binding assays suggest that the ability of PPARα to interact with the κB element in the *Il6* promoter strictly depends upon the sequence of the κB element and is enhanced in the presence of either p50 or RelA. Thus, PPARα binding to the κB element in the *Il6* promoter is promoted in the presence of a binding partner, namely p50 or RelA. Fourth, the presence of a minigene encoding RXRαD1, a decoy that interferes with the PPARα/NF-κB interaction, prevents upregulation of *Il6* in response to HFD consumption. Finally, unbiased ChIP-Seq analyses showed that PPARα and RelA/p50 have overlapping peaks near the κB site in the *Il6* promoter. The level of PPARα binding to the κB element in the *Il6* and *Tnfa* promoters was as high as to the authentic PPRE in the *Acox1* and *Cpt1b* promoters. These results support the idea that the PPARα/NF-κB heterodimer can stimulate transcription of *Il6* through binding to the κB element. It should be noted that PPARα binding to the κB element is sequence specific, and only a subset of κB elements, such as those in the *Il6* and *Tnfa* promoters, possess a PPARα-preferred binding sequence. Whether PPARα binds to the κB elements in the promoters of other genes needs to be tested in an unbiased manner.

How does PPARα have directionally opposite effects on NF-κB–mediated transcription in cardiomyocytes and other cell types? We found that the PPARα/NF-κB heterodimer induces transcriptional activation less potently than does the authentic NF-κB dimer. ChIP-Seq analyses showed that both endogenous PPARα and NF-κB bind to the κB element at baseline and during HFD consumption. However, NF-κB predominantly binds to the κB element in response to canonical stimuli. Under these conditions, overexpression of PPARα inhibits κB–mediated transcription, most likely by replacing the authentic NF-κB homodimer with the PPARα/NF-κB heterodimer, which activates transcription less potently than the NF-κB homodimer does. Thus, PPARα stimulates the κB element bound by the PPARα/NF-κB heterodimer in a PPARα ligand–dependent manner, whereas upregulation of PPARα may negatively affect stimulation of the κB element by the authentic NF-κB dimer. The timing and the mechanism that determine whether the κB element is controlled by the PPARα/NF-κB heterodimer or the NF-κB homodimer remain to be elucidated.

### Physiological role of PPARα–NF-κB–induced low-grade inflammatory reaction.

Several physiological roles of obesity-induced low-grade inflammation have been proposed. Obesity induces tissue damage due to metabolic and cellular stress. Low-grade inflammatory signaling may initially function as an adaptive response that promotes tissue repair and remodeling (59). A better understanding of the physiological significance of this pathway will be critical for its therapeutic exploitation, as it may enable the identification of patient populations in whom such interventions would be beneficial or detrimental, allow anticipation of potential side effects, and facilitate the development of strategies that selectively suppress pathological signaling while preserving beneficial functions.

### Study limitations.

Although the short-term HFD model enabled us to dissect the early molecular mechanism, it may not fully recapitulate cardiac remodeling observed during the chronic phase of obesity cardiomyopathy. In addition, PPARα possesses multiple functions in the heart, including transcription of genes involved in fatty acid oxidation. Thus, the significance of PPARα–NF-κB–mediated upregulation of inflammatory cytokines and other functions of PPARα during longer term obesity cardiomyopathy needs to be elucidated with time-dependent conditional downregulation of the PPARα–NF-κB pathway.

The molecular mechanism through which IL-6 mediates diastolic dysfunction remains to be elucidated. IL-6 downregulates Atp2a2 in cultured ventricular myocytes (60). Given that Atp2a2 mediates calcium reuptake into the sarcoplasmic reticulum, thereby facilitating cardiomyocyte relaxation, a decrease in Atp2a2 may contribute to impaired diastolic function. In addition, HFD consumption activates Stat3, a major downstream effector of IL-6, in a PPARα-dependent manner. JAK/Stat3 regulates cellular mechanisms relevant to cardiac fibrosis and increased stiffness (40). Although picrosirius red staining showed no overt cardiac fibrosis at the early phase (1 month of HFD consumption), this does not exclude the presence of subtle extracellular matrix remodeling may still occur below its detection limit. Even low levels of fibrosis undetectable by standard histology can increase myocardial stiffness and cause diastolic dysfunction (61). Thus, it is possible that IL-6 mediates diastolic dysfunction through regulation of cardiac fibrosis as well as myocardial stiffness.

### Sex differences in diastolic dysfunction.

In humans, women exhibit better insulin sensitivity and have less visceral fat and lower levels of circulating FFAs than men do (62). However, the prevalence of HFpEF is consistently higher in women than in men (63). Our study shows that PPARα functions dichotomously during HFD consumption: while PPARα supports metabolic resilience, it can also promote IL-6 expression. Whether sex-specific modification of PPARα exists and, if so, how it affects the PPARα–NF-κB pathway during obesity remain to be clarified.

### Therapeutic interventions for diastolic dysfunction.

Based on the molecular mechanism mediating the autocrine production of IL-6 in cardiomyocytes during HFD consumption, inhibiting PPARα in a cardiomyocyte-specific manner may prevent the development of diastolic dysfunction during the early phase of obesity. Because transcription of *Il6* in obesity is most likely initiated through fatty acid ligand binding to the preexisting PPARα–NF-κB complex on the *Il6* promoter, reducing the plasma level of fatty acids, by reducing obesity through lifestyle adjustments, should prevent upregulation of IL-6. Alternatively, more direct interventions targeting PPARα may be considered. It is important to note, however, that, because PPARα plays an essential role in fatty acid metabolism, a major source of energy in the heart, inhibiting every function of PPARα may cause undesirable side effects long term. Consistent with this notion, several *Ppara*-cKO mice exhibited systolic dysfunction at baseline, which was normalized upon HFD feeding (Figure 2B). We speculate that this reflects the critical role of fatty acid metabolism in maintaining basal cardiac energetics. Loss of PPARα may impair myocardial fatty acid utilization, resulting in systolic dysfunction in some animals, whereas increased fatty acid supply during HFD feeding may partially compensate for this defect and normalize cardiac function. Thus, specific interference with PPARα/NF-κB heterodimerization using the minigene derived from *Rxra* (RXRαD1) could be a better strategy for preventing early activation of the proinflammatory mechanism without affecting other potentially important functions of PPAR. Although we confirmed that the minigene does not interfere with the interaction between PPARα and RXRα, further investigation is required to evaluate the selectivity of the minigene against other interacting partners of PPARα. More accurate mapping of the PPARα-p50/RelA–interacting domain will be essential for the rational design of highly specific small-molecule antagonists that selectively disrupt this interaction. In addition, delivering the molecule inhibiting the PPARα/NF-κB interaction in a cardiomyocyte-specific manner, using nanoparticle approaches, should also prevent undesirable side effects.

In summary, we show that PPARα–NF-κB–mediated autocrine production of IL-6 plays an important role in mediating diastolic dysfunction during the early phase of obesity cardiomyopathy. A better understanding of the dichotomous functions of PPARα and specific molecular mechanisms mediating their effects should lead to specific and effective treatment to prevent the development of cardiomyopathy in patients with obesity.

## Methods

Detailed information on the materials and methods we used in this study are provided in Supplemental Methods.

### Sex as a biological variable.

We used mice of both sexes in all experiments. Cultured cardiomyocytes were prepared from mice and rats of both sexes.

### Statistics.

Normality was tested with the Shapiro-Wilk normality test. If the data exhibited a normal distribution, pairwise testing was performed with the Student’s *t* test, and multiple group comparisons were performed by 1-way ANOVA, followed by Tukey’s post-test. If the data failed normality testing, pairwise testing was performed with the nonparametric Mann-Whitney *U* test, and multiple group comparisons were performed with the nonparametric Kruskal-Wallis test, followed by Dunn’s post-test. *P* < 0.05 was defined as statistically significant. In figures, *P* < 0.05 is indicated by an asterisk; all error bars represent SEM.

### Study approval.

All animal procedures were reviewed and approved by the IACUC of Rutgers Biomedical and Health Sciences, Newark, New Jersey, USA.

### Data availability.

scRNA-Seq data are deposited in the National Center for Biotechnology Information Gene Expression Omnibus (NCI GEO) under accession code GE317479. ChIP-Seq data are deposited in NCBI GEO under accession code GE316422. Supporting data values are provided in Supplemental Data. For other experimental procedures, see Supplemental Methods.

## Author contributions

SO designed the study, performed most of the experiments, analyzed data, and interpreted results. KBS maintained animal lines fed an HFD. SB performed histological analyses. KBS, AC, and SIK performed Western blot analyses. EAS obtained and analyzed the scRNA-Seq data. PZ performed PV loop analyses. AS, TY, YI, WM, and SI performed echocardiographic measurements and analyses. JB purified recombinant proteins. JF and DPD performed FACS analyses. JP and YJC performed bioinformatics analyses of ChIP-seq data. MT and EAS isolated adult cardiomyocytes and fibroblasts from mice. MN performed RNA-seq experiments using H9c2 cells. SO and JS conceived the ideas and wrote the manuscript. JS oversaw the entire study and maintains experimental resources and environment. All authors reviewed and approved the manuscript for publication.

## Funding support

This work is the result of NIH funding, in whole or in part, and is subject to the NIH Public Access Policy. Through acceptance of this federal funding, the NIH has been given a right to make the work publicly available in PubMed Central.

- The New Jersey Health Foundation (grant PC-56-16).
- American Heart Association (AHA) Transformational Project Award 19TPA34850170 (to SO).
- US Public Health Service grants HL91469, HL102738, HL112330, HL138720, HL144626, and HL150881.
- AHA Merit Award 20 Merit 35120374 (to JS).
- The Fondation Leducq Transatlantic Network of Excellence (grant 15CVD04 to JS).

## Supplementary Material

Supplemental data

Unedited blot and gel images

Supporting data values

## Version 1 02/12/2026

In-Press Preview

## Version 2 04/01/2026

Electronic publication
