# Supplementary material for: PPARα–NF-κB heterodimer mediates obesity-induced diastolic dysfunction through autocrine production of IL-6
Source: J Clin Invest. 2026 Feb 12;136(7):e196238. doi: 10.1172/JCI196238 (PMC13038213; doi:10.1172/JCI196238)
Supplement: Supplemental data [file jci-136-196238-s279.pdf]

**Supplementary Materials**

# PPARα-NFκB heterodimer mediates obesity-induced diastolic dysfunction through autocrine production of IL-6

Shin-ichi Oka[1], Eun-Ah Sung[1], Peiyong Zhai[1], Kevin Schesing[1], Santosh Bhat[1], Adave Chin[1], Jiyeon Park[2], Yeun-Jun Chung[2], Akihiro Shirakabe[1], Takanobu Yamamoto[1], Yoshiyuki Ikeda[1], Wataru Mizushima[1], Shohei Ikeda[1], Mingming Tong[1], Jaemin Byun[1], Michinari Nakamura[1], Samuel Kim[1], Jamie Francisco[1], Dominic P Del Re[1] and Junichi Sadoshima[1]

Affiliation:
[1]Rutgers New Jersey Medical School Department of Cell Biology and Molecular Medicine, Rutgers Biomedical and Health Sciences, Newark, NJ 07101.
[2]Precision Medicine Research Center, College of Medicine, The Catholic University of Korea, Seoul, Republic of Korea.

Running Title: PPAR-induced IL-6 mediates obesity cardiomyopathy

Correspondence:
Junichi Sadoshima, MD PhD
Cardiovascular Research Institute,
Rutgers Biomedical and Health Science
185 South Orange Ave., MSB G609
Newark, NJ 07103
Tel: (973)972-8619
Fax: (973)972-8919
Email: sadoshju@njms.rutgers.edu

**Supplementary Methods**

**Animal experiments**

Cardiac specific Tg-*Ppara*, line 404-3, was provided by Drs. Daniel P. Kelly and Teresa Leone at the Sanford-Burnham Medical Research Institute. Systemic *Ppara* knockout and *Ribo-tag* mice were purchased from Jackson Laboratory. *Pparaflox/flox* and *Il6flox/flox* mice were purchased from Institute Clinique de la Souris and the CNB Mouse Cryopreservation facility, respectively. *Myh6-Cre* transgenic mice were kindly provided by Dr. Jeffery Molkentin at the University of Cincinnati. *Pparaflox/flox*, *Il6flox/flox* and *Ribo-tag* mice were crossed with *Myh6-Cre* mice to generate cardiac-specific *Ppara* knockout, *Il6* knockout and *Ribo-tag* mice. Both genders of mice were used in this study. Plasma levels of IL-6 were determined with Quantikine ELISA (R & D systems). The investigators were blind to the treatment and genotype of the animals. All procedures involving animals were performed in accordance with protocols approved by Rutgers Biomedical and Health Sciences.

**HFD consumption**

Both male and female mice aged 8-10 weeks were fed ad libitum with HFD (Research Diets D12492) for 1-4 months. A control group of mice matched for age and gender were fed a control diet (ND) for the same period of time. Changes in body weight during ND (Research Diet 5053) and HFD consumption were evaluated every 4 weeks. We excluded mice that did not gain 20% of body weight after being fed HFD for 2 months from the subsequent analysis. There were no exclusions among mice fed the ND.

**Pressure-volume (PV) measurements and analysis**

PV measurements and analysis were performed using the MPVS Ultra Single Segment system (Millar Instruments) under the control of the LabChart Pro (v8.1.30, AD Instruments, Colorado Springs, Colorado) coupled with MPVS Ultra Control Interface (Millar Instruments Inc). After anesthesia with pentobarbital (60-80 mg/kg i.p.) or avertin (300 mg/g i.p.), the right carotid artery was cannulated with a high-fidelity mouse PV catheter transducer (1.0-F, Model PVR-1030, Millar Instruments), which was advanced into the left ventricular (LV) cavity. The PV relationship was continuously recorded. After stabilization, the end-systolic PV relationship (ESPVR) and end-diastolic PV relationship (EDPVR) were obtained by varying end-diastolic volume through intermittent occlusion of the inferior vena cava. The PV loop data analysis was done offline using the LabChart Pro software. On average, 10-20 loops were analyzed. The ESPVR was obtained using a linear ($P_{es}=E_{es}*V_{es}+B$) fitting model, where the end-systolic elastance $E_{es}$ indicates systolic function. The EDPVR was obtained using an exponential ($P_{ed}=C*exp^{\beta Ved}$) fitting model, where the chamber stiffness constant $\beta$ indicates diastolic function (1). In order to minimize bias, the investigators were blind to the treatment and genotype of the animals.

**Echocardiography**

Transthoracic echocardiography was performed as previously described (2), using a high-resolution Micro-Ultrasound system (Vevo 3100, FUJIFILM VisualSonics Inc., Toronto, Canada) or an ultrasonography system (Acuson Sequoia C256; Siemens Medical Solutions), depending on the experimental cohort. Mice were anesthetized with

either 12 µl/g body weight of 2.5% Avertin (Sigma-Aldrich) or maintained under continuous inhalation of 0.75–1% isoflurane. The chest was shaved, and electrocardiographic leads were attached to each limb using needle electrodes. A 13-MHz linear ultrasound transducer or a high-frequency probe (Vevo 3100) was used to acquire two-dimensional and M-mode images from the parasternal short-axis view at the mid-papillary muscle level. M-mode tracings were recorded at a sweep speed of 100–200 mm/s. Measurements of left ventricular internal dimensions were obtained from three or more cardiac cycles and averaged. LV end-diastolic diameter (LVEDD or LVIDd) was measured at the time of maximal diastolic dimension, and LV end-systolic diameter (LVESD or LVIDs) at the time of maximal systolic contraction. LV ejection fraction and fractional shortening were calculated as follows: Ejection fraction=$[(LVEDD)^3-(LVESD)^3]/(LVEDD)^3$ x 100; Fractional shortening=(LVEDD-LVESD)/LVEDD x 100. All image acquisition and measurements were performed by investigators blinded to the animal genotype and treatment group.

**Anti-IL-6R antibody**

Rat anti-mouse IL-6R antibody (MR16-1) was kindly provided by Chugai Pharmaceuticals CO. Control Rat IgG was purchased from Sigma (I4131). After 1 week of HFD consumption, these antibodies (0.5 mg/mouse) were intraperitoneally injected every week. PV loop analyses were performed after 1 month of HFD consumption.

**Histochemical analysis**

Heart specimens were fixed in 10% neutral-buffered formalin for 24 hours, dehydrated through graded ethanol, and embedded in paraffin. Serial sections were prepared at a thickness of 6 μm and mounted on glass slides. Interstitial fibrosis was evaluated by staining with picrosirius red (PSR), which selectively binds to collagen fibers. After staining, sections were rinsed, dehydrated, and coverslipped with mounting medium. Images were captured under bright-field microscopy using identical exposure settings for all samples. The fibrotic regions, defined as PSR-positive (red-stained) areas, were quantified using ImageJ software (NIH, Bethesda, MD).

**Plasmids**

The mammalian expression vectors for PPARα and PPARαΔAF2 have been described previously (3). The luciferase reporter gene driven by 3xPPRE, pPPRE-tk-luc, was kindly provided by Dr. Ronald Evans at the Salk Institute. The reporter genes driven by the mouse *Il6* promoter were generated by insertion of 1 or 0.6 Kb of the promoter region into pGL3basic (Promega). The 1 Kb promoter region was amplified with oligonucleotide primers (5' to 3'):
AAAAAAAGATCTAGCTAAGATACAATGAGGTCCTTC and
ATATATAAGCTTTCTGGAATTGACTATCGTTCTTGGTG. The 0.6 Kb promoter region was amplified with oligonucleotide primers (5' to 3'):
AAAAAAAGATCTGTTGTCCAGGTTGGGTGCT and
ATATATAAGCTTTCTGGAATTGACTATCGTTCTTGGTG. C-terminal Flag-tagged RelA (pcDNA3-RelA-Flag) and p50 (pcDNA3-p50-Flag) were gifts from Dr. Stephen Smale (Addgene #20012 and #20018). Bacterial expression vectors for GST-fused RelA

(pCold-GST-RelA) and p50 (pCold-GST-p50) were generated by standard plasmid construction strategies. The bacterial expression vectors pCold-GST-PPARα, pCold-GST-RXRα and pCold-GST-RXRαD1 were described previously (3). The RXRαD1 region with N-terminal Myc tag was inserted into the AAV shuttle vector to generate AAV-Myc-RXRαD1. The cDNA of *RXRα* corresponding to amino acids 345-422 was inserted into pCold-GST-HA to generate pCold-GST-HA-RXRαD2.

**Triglyceride content**

Frozen left ventricles (LVs) were weighed, and tissue was extracted at a fixed ratio of 250 μL of 5% Triton X-100 per 50 mg of tissue. The homogenates were then heated at 80 °C for 5 minutes and slowly cooled to room temperature. After centrifugation (1,300 × *g*, 10 minutes), triglyceride content in the supernatant was measured using the Triglyceride Quantification Kit according to the manufacturer's instructions (BioVision, Cat. #EGA-200).

**Adenovirus Vectors**

Recombinant adenoviruses expressing Myc-tagged PPARα and Myc-PPARαΔAF2 were generated using the AdMax™ adenoviral vector system (Microbix Biosystems) according to the manufacturer's protocol with standard optimizations. Expression cassettes encoding either full-length Myc-tagged PPARα or the AF2 domain–deleted mutant (ΔAF2) were subcloned into the pDC316 shuttle vector, which enables homologous recombination with the adenoviral backbone during co-transfection. To produce recombinant viral particles, HEK-293 cells, which stably express adenoviral E1

genes, were co-transfected with either pDC316-Myc-PPARα or pDC316-Myc-PPARαΔAF2 together with the pBHG vector using Lipofectamine 2000 (Invitrogen). The HEK-293 cells were originally obtained from an academic source and have been maintained in our laboratory for many years. When cytopathic effects (CPE) became evident, cells were harvested in virus storage buffer (20 mM Tris-HCl, pH 8.1, 25 mM NaCl, 2.5% glycerol) and subjected to three cycles of freeze–thawing to release intracellular virus particles. The lysates were then centrifuged at 11,000 × g for 10 minutes at 4°C to remove cellular debris. The clarified supernatant containing crude adenovirus was collected, aliquoted, and stored at –80°C, and was used directly as viral stock for subsequent infection experiments without further purification.

**Luciferase reporter gene assay**

Luciferase assays were performed in primary cultured rat cardiomyocytes as previously described (see details in the online supplement). Reporter plasmids (0.3 μg per well), such as *Il6* promoter-luc, mammalian expression vectors (0.7 μg per well), such as pDC316-PPARα, and siRNA (20 pM) against *PPARα* (Qiagen SI01963451) were transfected into cells plated in 12 well plates using LipofectAmine 2000 (Invitrogen). Total plasmids were kept at 1 μg per well using pDC316 control vector. The total siRNA was kept at 20 pM per well using control siRNA. Two μl LipofectAmine 2000 was used per well. Fifty μl OPTI-MEM was used for dilution of each plasmid vector and of LipofectAmine 2000. For fatty acid stimulation, cells were treated with palmitic acid (PA; 100 or 300 μM) or oleic acid (OA; 150 or 500 μM) for 16 hours. Where indicated, cells were treated with

the PPARα agonist WY14643 (1 or 10 μM) for 16 hours. Luciferase activities were measured 2–5 days after transduction using a luciferase assay system (Promega).

**Proximal ligation assay (PLA)**

In situ proximity ligation assay (PLA) was conducted using the Duolink® In Situ Red Starter Kit Mouse/Rabbit (MilliporeSigma, Cat# DUO92101), following the manufacturer's instructions with minor modifications. Primary cultured neonatal rat ventricular cardiomyocytes were seeded onto gelatin-coated coverslips and cultured under standard conditions. Cells were fixed with 4% paraformaldehyde (PFA) in phosphate-buffered saline (PBS) for 20 minutes at room temperature, followed by three washes with PBS. Permeabilization was performed using 0.1% Triton X-100 in PBS for 10 minutes. After washing, the cells were blocked with Duolink® Blocking Solution for 1 hour at 37°C in a humidified chamber. After blocking, cells were incubated overnight at 4°C with rabbit anti-PPARα (Cayman Chemical, Cat# 101720) and mouse anti-RelA (Cell Signaling Technology, Cat# 8242) primary antibodies diluted in Duolink® Antibody Diluent. The next day, cells were washed with Wash Buffer A and incubated with PLA probes (anti-rabbit PLUS and anti-mouse MINUS) for 1 hour at 37°C. Ligation was carried out at 37°C for 30 minutes using the ligation enzyme and buffer provided in the kit. Subsequently, rolling circle amplification was performed for 100 minutes at 37°C using polymerase and fluorescently labeled detection oligonucleotides, generating discrete red fluorescent puncta indicative of protein-protein proximity. After amplification, cells were washed with Wash Buffer B, counterstained with DAPI, and

mounted using Duolink® In Situ Mounting Medium with anti-fade reagent. Images were acquired using a fluorescence microscope.

**Adeno-associated virus (AAV) vectors**

Shuttle vectors of AAV-DJ/8-GFP (AAV-control) and AAV-DJ/8-Myc-RXRαD1 (AAV-Myc-RXRαD1) were used to generate AAV vectors at the AAV core facility of Rutgers University. The backbone vector (pAAV-MCS) was purchased from Cell Biolabs. HEK293 cells (Cell Biolabs) were cotransfected with the shuttle vector, pAAV-DJ/8 vector, and helper plasmid at a 1:1:1 ratio using polyethylenimine. The AAV vectors were purified by the iodixanol gradient/ultracentrifugation method, and the AAV fraction was concentrated using a VIVASPIN 20 concentrator (100 kDa cutoff, Sartorius). The virus titer was determined using the Cell Biolabs AAV quantitation kit (VPK-145). Doses of $5 \times 10^{11}$ vectors were injected i.v. via the jugular vein as described previously (4).

**Quantitative Real Time PCR**

Total RNA was prepared from left ventricles using the RNeasy Fibrous Tissue Mini Kit (Qiagen), and then cDNA was generated using M-MLV Reverse transcriptase (Promega). Real-time RT-PCR was performed using the Maxima SYBR Green qPCR master mix (Fermentas). RPS15 was used as an internal control. The mean value from WT mice was expressed as 1. PCRs were carried out using the following oligonucleotide primers (5' to 3'): *Atp2a2*, TCTTGATCCTCTACGTGGAACCTTTGC and GTTGTTCCAGGTAGTTTCGGGCCA; *Myh7*, AAGGTGAAGGCCTACAAGCGC and AGCTTGTTGACCTGGGACTCG; *Mmp2*, AATGCCATCCCTGATAACCTGGATG and

AACAGGGCCAGCTCAGCAGC; *Col1a1*, CAAGAACAGCGTAGCCTACATGGA and TCTTGCCCCAAGTTCCGGTGT; *Ccl5*, CTGCTTTGCCTACCTCTCCCT and CTCCAAATAGTTGATGTATTCTTGAACCC; *Il-1α*, AAAGACGGCACACCCACCCTG and GTGCTGATGTACCAGTTGGGGAA; *Il-1β*, AAAGACGGCACACCCACCCTG and GTGCTGATGTACCAGTTGGGGAA; *Il6 (mouse)*, GTCCTTCCTACCCCAACTTCCAATG and GCTTAGGCATAGCACACTAGGTTTG; *Il6 (rat),* GTCCTTCCTACCCCAATTTCCAATG and GCTTAGGCATAACGCACTAGGTTTG; *Mcp1*, CACTCACCTGCTGCTACTCATTCA and CTCTCTCTTGAGCTTGGTGACAAA; *Mif*, GCCTGCTGTCCGATCGCCT and AAGAACAGCGGTGCAGGTAAGTG; *Mip1a*, GACTGCCTGCTGCTTCTCCTA and TCAGTGATGTATTCTTGGACCCAGG; *Mip1b*, CTTTGTGATGGATTACTATGAGACCAG and GAGCTGCTCAGTTCAACTCCAAGT; *Mip2*, TGACTTCAAGAACATCCAGAGCTTG and TCCTTTCCAGGTCAGTTAGCCTTG; *Tnfa*, TTCCAGCTGGAGAAGGGGGAC and TCACAGAGCAATGACTCCAAAGTAGA; *Acox1*, ATGAATCCCGATCTGCGCAA and TTCTCGATTTCTCGACGGCG; *Cd36*, GAATCTGAAGAGACCTTACATTGTACC and CACTCCAATCCCAAGTAAGGCCAT; *Ech1,* CTGACGAGGCCCTGGACAGT and TGATTTTTGACCCCTGCACAGCCA; *Fabp3*, AAGGAGGCGTGACCTGGCTG and ACCTTGGAGCACCCTTTGGATACA; *Mcad*, GAAGCTGATGAGGGACGCCA and GCTTGGAGCTTAGTTACACGAGG; *Pdk4*, GCCTGGGCCATCCTTCCTCTC and GTATGTGTGTCCTACAGAAGCGC; *Rps*15, TTCGCAAGTTCACCTACC and CGGGCCGGCCATGCTTTA.

**Chromatin immunoprecipitation (ChIP) assay**

Heart tissue or cultured cardiomyocytes were cross-linked with formaldehyde. The nuclear fraction was isolated and sonicated to generate a chromatin solution that was then used for immunoprecipitation. Primers used for investigating the mouse genome are as follows: (5' to 3'): *Il6*– TTAATAAGGTTTCCAATCAGCCCCAC and AGAGCAGAATGAGCTACAGACATCC; *Acox1* – GGAAAGATCACGTGAACCTGGAG and TCCTCACGTGACCGGCTGCAAT; *Fatp1* – GAATTCAAAGTCTCAGGTCAACCCTTG and CTCTGCCCCCACCCCCATAAA; *Pdk4* – TGTAAACAAGGACAAGTCTGGGCG and AAGGGGCAAAGGGTGAGAGGGAG. The collected chromatin fragments were validated by qPCR with Maxima SYBR Green qPCR master mix (Fermentas).

**ChIP-sequencing**

Chromatin immunoprecipitation sequencing (ChIP-seq) was outsourced to Active Motif, Inc. (Carlsbad, CA), which carried out the experiment following their standardized protocols. For each experimental condition, left ventricular tissues from three mouse hearts were pooled to prepare a chromatin solution. The tissues were cross-linked with 1% formaldehyde to preserve protein-DNA interactions, followed by nuclear extraction and sonication to shear chromatin into fragments averaging 200–500 base pairs in length. Immunoprecipitation was conducted using antibodies against PPARα (Cayman Chemical, #101720), RelA (Active Motif, #39369), and p50 (Cell Signaling Technology, #3035), with isotype-matched IgG controls included to assess specificity. After reverse cross-linking and purification of immunoprecipitated DNA, libraries were prepared and sequenced on an Illumina platform. Peak calling and alignment to the mouse genome

(mm10) were performed using proprietary bioinformatics pipelines provided by Active Motif. ChIP-seq data were visualized and analyzed using the Integrated Genome Browser (IGB).

**Primary cultures of neonatal rat ventricular myocytes and fibroblasts**

Primary cultures of ventricular cardiac myocytes and fibroblasts were prepared from 1-day-old Crl:(WI) BR-Wistar rats (Harlan). Cardiac myocyte- and fibroblast-rich fractions were obtained by centrifugation through a discontinuous Percoll gradient. Cells were cultured in complete medium (CM) containing Dulbecco's modified Eagles's medium/F12 supplemented with 5% horse serum, 4 μg/ml transferrin, 0.7 ng/ml sodium selenite, 2 g/l bovine serum albumin (fraction V), 3 mM pyruvate, 15 mM Hepes pH 7.1, 100 μM ascorbate, 100 mg/l ampicillin, 5 mg/l linoleic acid, and 100 μM 5-bromo-2'-deoxyuridine (Sigma). Culture dishes were coated with 0.3% gelatin.

**Immunoblotting**

Mouse heart tissue was dissected and the left ventricle was lysed in a lysis buffer containing 50 mM Tris pH 7.4, 150 mM NaCl, 50 mM NaF, 10 mM EDTA, 1% Triton-X 100, 0.1 mM DTT and 1x Protease Inhibitor Cocktail (Sigma). Total protein lysates (10–30 μg) were incubated with SDS sample buffer [Final concentration: 100 mM Tris pH 6.8, 2% SDS, 5% glycerol, 2.5% 2-mercaptoethanol, and 0.05% bromophenol blue] at 95°C for 5-20 minutes. The denatured protein samples were transferred to polyvinylidene difluoride membranes and probed with antibodies against PPARα (Cayman 101710), RXR (Santa Cruz ΔN197), RelA (Cell Signaling Technology (CST)

8242), p50 (CST 13586), Phospho-Stat3Y705 (CST 9145), Stat3 (CST 12640), IL-6 (Abcam Ab229381), Myc (CST 2272), CD68 (Santa Cruz H-255), Troponin T (Abcam Ab10214) and Tubulin (Sigma T6199).

**Purification of bacterially expressed proteins**

The BL21 *E.coli* strain was transformed with pCold-GST-PPARα, pCold-GST-RelA, pCold-GST-p50, pCold-GST-RXRα, pCold-GST-RXRαD1 and pCold-GST-RXRαD2. The *E.coli* was grown in 3 ml LB medium overnight at 37°C and then transferred to 250 ml LB medium. After overnight culture at 15°C, the *E.coli* was lysed in lysis buffer (1% Triton X-100, 1 mM dithiothreitol (DTT), PBS) with sonication. The lysate was incubated with 0.5 ml Glutathione-sepharose 4B (GE Healthcare) for 1 hour at 4°C. The sepharose was washed 3 times with 5 ml lysis buffer and then suspended with 1 ml cleavage buffer (20 mM Tris pH 7, 150 mM NaCl, 1 mM DTT) containing 200 units/ml of PreScission protease (GE Healthcare). After overnight incubation at 4°C, the supernatant was collected as recombinant PPARα. The recombinant PPARα was stored at -80°C.

***In vitro* pulldown assay**

Recombinant proteins and/or biotin-labeled double-stranded DNAs were incubated with 150 μl binding buffer [10 mM Hepes pH 7.9, 2.5 mM $MgCl_2$, 50 mM KCl, 150 mM NaCl, 5% Glycerol, 1 mM DTT, 0.1% NP-40 substitute] with GSH beads or streptavidin beads at 4°C for 2 hours. The beads were washed with 1 ml binding buffer 3 times. The precipitates were eluted with sample buffer [100 mM Tris pH 6.8, 2% SDS, 5% glycerol,

2% 2-Mercaptoethnol, 0.05% bromophenol blue] and subjected to Western blot analyses.

Double-stranded DNA used for the pulldown assays were as shown below. Biotin was conjugated to the following forward oligonucleotides. Non-biotinylated reverse oligonucleotides were annealed with the indicated oligonucleotides.

| Genes | Sequences (5'-3') |
|---|---|
| PPRE: | AAATAAATGTAGGTCAAAGGTCACAGGAGC |
| PPREm: | AAATAAATGTAAATCAAAAATCACAGGAGC |
| *Acox1*: | CGAAAGGGTAACAGGACAAAGGTTACGTTCG |
| *Cd36*: | TCCCATCCAAGTAAGTCAGAGGCCAGAGAAC |
| *Cpt1b*: | ATAGCTGAATGTAGGGAAAAGGTCACCAGGA |
| DR3: | GAGTTGCCAGGTGGGTAAAGTGGGTGAAGTACAGAA |
| *Ccl5*: | TTTTGTGGAAACTCCCCAAGTCCTGGGGCTGGGGG-GGGGCACTTCCTCTGCA |
| *Il1a*: | AAGAGCAAAGTAGGGTCATTGCCCTTGCAAGCACG |
| *Il1b*: | TTAAAAATATTGGGTTTTCCTGGGTTAGCTGTACA |
| *Il6*: | TTTTATCAAATGTGGGATTTTCCCATGAGTCTCAAA |
| *Tnfa:* | ATGGGATGAGTATGGGGCAGCCCCAGAGGGAATGAAC |
| *Mip1b*: | AGTCCCTATAAAGAGGGGTTCCCAACTCAGATCAGC |
| *Mip2*: | GACCCTGAGCTCAGGGAATTTCCCTGGTCCCCGGGCTT |
| *Il6m1*: | TTTTATCAAATGTGGAATTTTCCCATGAGTCTCAAA |
| *Il6m2*: | TTTTATCACGCGTGGGATTTTCCCATGAGTCTCAAA |

*Il6m3*: TTTTATCAAATGTGGGATTTTTTATGAGTCTCAAA

*Il6m4*: TTTTATCAAATGTAGGATTTTCCCATGAGTCTCAAA

*Il6m5*: TTTTATCAAATGTAGGTCATTCCCATGAGTCTCAAA

**Single cell RNA sequencing**

C57BL/6J mice were fed with HFD for 4 weeks, while control mice were maintained on a ND. Adult mouse heart cells were isolated from ND-fed (n = 3) and HFD-fed (n = 3) mice using previously published protocols (5). Freshly isolated heart cells were pooled into ND and HFD groups and immediately fixed using the Evercode Fixation v2 kit (Parse Biosciences) according to the manufacturer’s instructions. Briefly, cells were resuspended in Cell Prefixation Buffer, filtered to obtain a single-cell suspension, and incubated with Cell Fixation Solution for 10 minutes on ice. Following permeabilization for 3 minutes on ice, the reaction was neutralized with Cell Neutralization Buffer and cells were collected by centrifugation and resuspended in Cell Buffer. Fixed cells were processed into two sublibraries using the Evercode WT Mini v3 kit (Parse Biosciences). Combinatorial barcoding, cDNA synthesis, amplification and library preparation were performed according to the manufacturer’s protocols. Barcoded libraries were sequenced on an Illumina NovaSeq 6000 platform using the NovaSeq 6000 SP Reagent Kit v1.5 (200 cycles). Base cells were demultiplexed and processed with the Parse Biosciences Whole Transcriptome pipeline. Reads were aligned to the mouse reference genome (mm10) and digital gene expression (DGE) was generated. DGE matrices were analyzed in Trailmaker, Parse’s cloud-based analysis platform. Standard preprocessing steps, including quality control, normalization, dimensionality

reduction, clustering, and cell type annotation, were performed within the Trailmaker interface. Differential gene expression analysis and data visualization were conducted in Trailmaker using built-in statistical tools. KEGG pathway enrichment analysis was performed using ShinyGO 0.82 (6).

## Supplemental References


1. Burkhoff D, Mirsky I, and Suga H. Assessment of systolic and diastolic ventricular properties via pressure-volume analysis: a guide for clinical, translational, and basic researchers. *American journal of physiology Heart and circulatory physiology.* 2005;289(2):H501-12.

2. Oka S, Alcendor R, Zhai P, Park JY, Shao D, Cho J, et al. PPARalpha-Sirt1 Complex Mediates Cardiac Hypertrophy and Failure through Suppression of the ERR Transcriptional Pathway. *Cell metabolism.* 2011;14(5):598-611.

3. Oka SI, Zhai P, Yamamoto T, Ikeda Y, Byun J, Hsu CP, et al. PPARalpha Association With Sirt1 Suppresses Cardiac Fatty Acid Metabolism in the Failing Heart. *Circulation Heart failure.* 2015.

4. Kashihara T, Mukai R, Oka S, Zhai P, Nakada Y, Yang Z, et al. YAP mediates compensatory cardiac hypertrophy through aerobic glycolysis in response to pressre overload. *J Clin Invest.* 2022;132:e150595.

5. Ackers-Johnson M, Li PY, Holmes AP, O'Brien SM, Pavlovic D, and Foo RS. A Simplified, Langendorff-Free Method for Concomitant Isolation of Viable Cardiac Myocytes and Nonmyocytes From the Adult Mouse Heart. *Circ Res.* 2016;119(8):909-20.

6. Ge SX, Jung D, and Yao R. ShinyGO: a graphical gene-set enrichment tool for animals and plants. *Bioinformatics.* 2020;36(8):2628-9.

**Supplementary Data**

**Table S1.**
**Organ weight data of *Il6* cKO mice under HFD consumption.**

| | Wild ND(16) | Wild HFD (19) | cKO ND (11) | cKO HFD (12) |
|---|---|---|---|---|
| Initial Body weight (BW), g | 28.09±6.12 | 25.05±6.26 | 27.18±7.46 | 26.00±3.7 |
| Final Body weight (BW), g | 31.38±5.4 | 38.53±10.12 | 31.92±5.99 | 37.52±8.29 |
| Tibia length (TL), mm | 18.52±0.85 | 19.82±1.71 | 18.10±0.84 | 18.33±0.47 |
| Heart weight (HW), mg | 136.1±23.64 | 127.94±25.33 | 125.9±23.52 | 129.33±18.31 |
| LV weight (LVW), mg | 100.12±27.15 | 93.15±23.21 | 103.3±91.54 | 87.50±10.87 |
| Lung weight (LUW), mg | 166.25±4.54 | 165.68±25.76 | 163.90±20.04 | 160.83±13.83 |
| HW/TL | 7.37±1.4 | 6.44±1.03 | 6.98±1.47 | 7.04±0.88 |
| LVW/TL | 5.43±1.5 | 4.66±0.90 | 5.08±1.21 | 4.76±0.55 |
| LUW/TL | 9.00±1.17 | 8.39±0.95 | 9.08±1.32 | 8.77±0.69 |

High-fat diet (HFD) feeding was conducted for 1 month. Mice of both sexes, approximately 2–5 months of age, were used in all groups. Data are mean ± SD. LV: Left Ventricle. Statistical significance was determined with Student's *t* test in Initial and final body weight HFD, Tibia length HFD, LV weight ND, Lung weight ND, HW/TL HFD, LVW/TL ND, LUW/TL ND, and LUW/TL HFD, and the Mann–Whitney U test for all other comparisons. * $p<0.05$ vs Wild. Parenthesis indicates N.

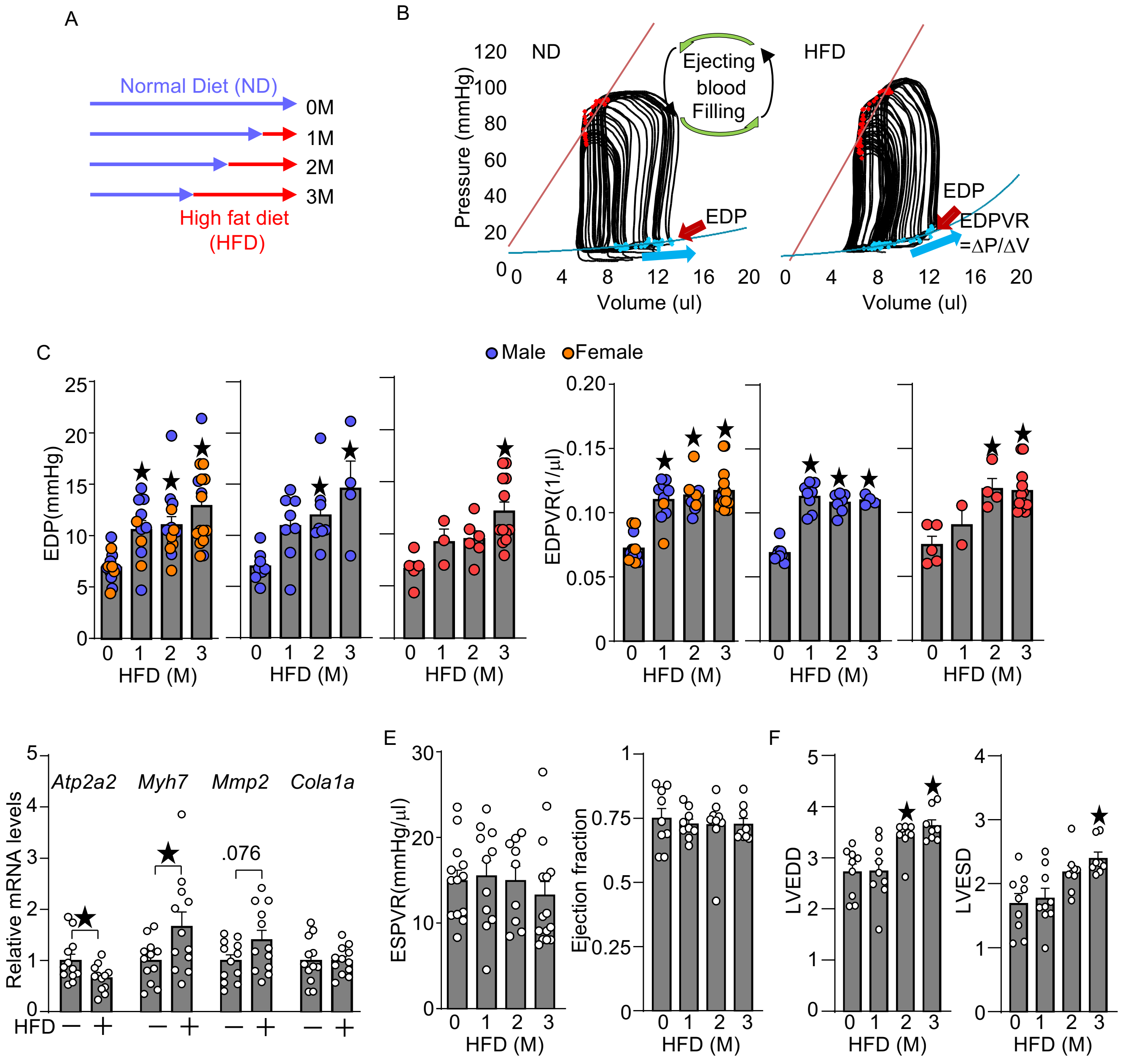


**Figure S1.** HFD induces diastolic dysfunction. (A) Schematic representation of HFD consumption schedule. Similarly aged wild-type mice were examined with PV loop analysis and echocardiographic measurements. (B) Representative PV loop results after 1 month of HFD consumption. (C) Increased end-diastolic pressure (EDP) and end-diastolic pressure–volume relationship (EDPVR) were observed after 1 to 3 months of HFD consumption in wild-type mice, shown separately for male and female groups as well as combined data. (D) Expression of diastolic heart failure–related genes (*Atp2a2, Myh7, Mmp2*, and *Col1a1*) in the hearts of wild-type mice after 1 month of HFD feeding. (E) Cardiac systolic function was preserved under HFD consumption conditions. (F) Effect of high-fat diet on cardiac chamber size in wild-type mice. High-fat diet did not markedly affect chamber dimensions after 1 month of feeding. The numbers of mice examined in each experimental group were: 9-16 (C), 12 (D), 9-15 (E) and 8-9 (F). Statistical significance is indicated by an asterisk and was determined using the Kruskal–Wallis test for EDP (both males and females) and EDPVR (females) in panels (C) and (F), Student's *t*-test in (D), and one-way ANOVA for all other comparisons.

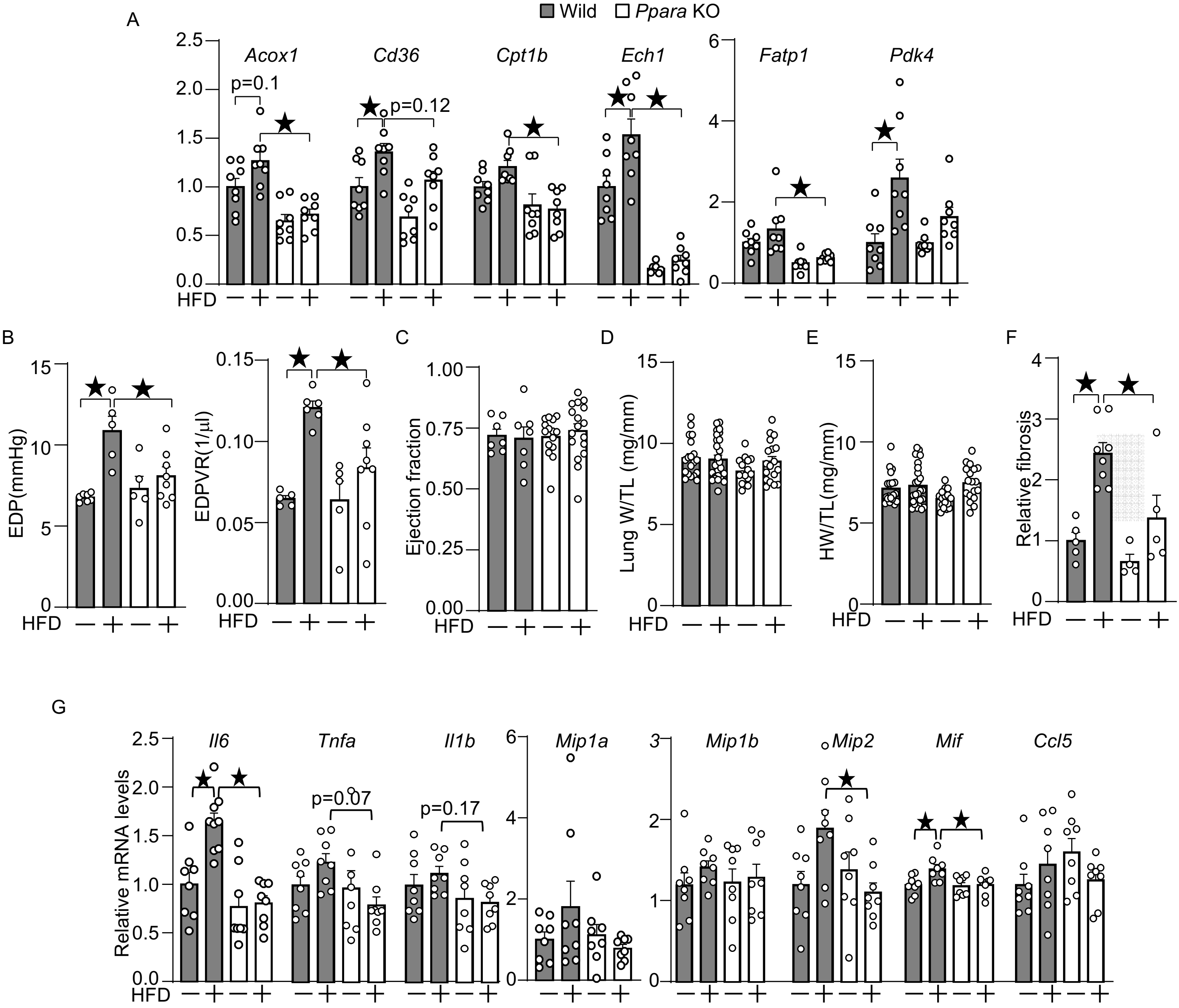


**Figure S2.** PPARα mediates gene expression changes and diastolic dysfunction during HFD consumption. (A) HFD-induced PPARα target gene expression is inhibited in *Ppara* KO mice. Expression levels of indicated PPAR target genes after 1 month of HFD consumption were examined in *Ppara* KO mice. (B) HFD-induced diastolic dysfunction is ameliorated in *Ppara* KO mice. (C) After 1 month of HFD feeding, cardiac systolic function, evaluated via ejection fraction, was not changed in *Ppara* KO mice. (D) After 1 month of HFD feeding, lung congestion, characterized by Lung weight/Tibia length ratio, was not changed in *Ppara* KO mice. (E) After 1 month of HFD feeding, cardiac hypertrophy, evaluated via heart weight/tibia length ratio, was not changed in *Ppara* KO mice. (F) HFD-induced cardiac fibrosis was ameliorated in *Ppara* KO mice. (G) Expression levels of indicated cytokines after 1 month of HFD consumption in *Ppara* cKO mice. The numbers of mice examined in each experimental group were: 8 (A), 5-11 (B), 7-17 (C), 16-27 (D), 16-27 (E), 4-8 (F) and 8 (G). Statistical significance is indicated by a star and was assessed using the Kruskal–Wallis test for *Fatp1* and *Pdk4* in (A), (C), (D) and (E), and for *Tnfa* in (G), and ANOVA for all other comparisons.

Figure S3

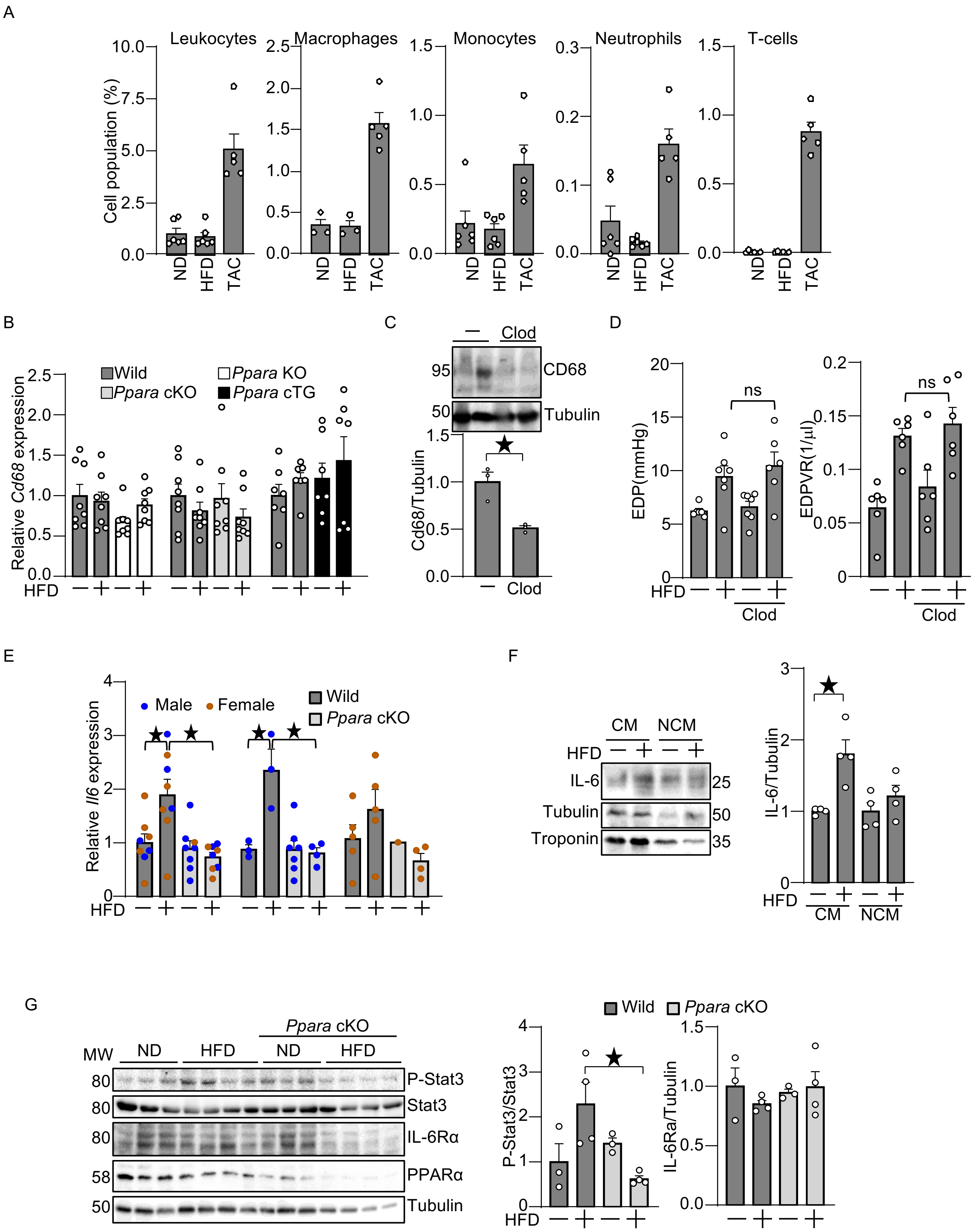
A
Leukocytes
Macrophages
Monocytes
Neutrophils
T-cells
Cell population (%)
ND
HFD
TAC
B
Wild
Ppara KO
Ppara cKO
Ppara cTG
Relative Cd68 expression
HFD
C
Clod
CD68
Tubulin
95
50
Cd68/Tubulin
D
ns
EDP(mmHg)
EDPVR(1/μl)
E
Male
Female
Wild
Ppara cKO
Relative Il6 expression
F
CM
NCM
IL-6
Tubulin
Troponin
25
50
35
IL-6/Tubulin
G
Ppara cKO
MW
ND
HFD
80
58
50
P-Stat3
Stat3
IL-6Rα
PPARα
Tubulin
P-Stat3/Stat3
IL-6Ra/Tubulin

Figure S3 continued

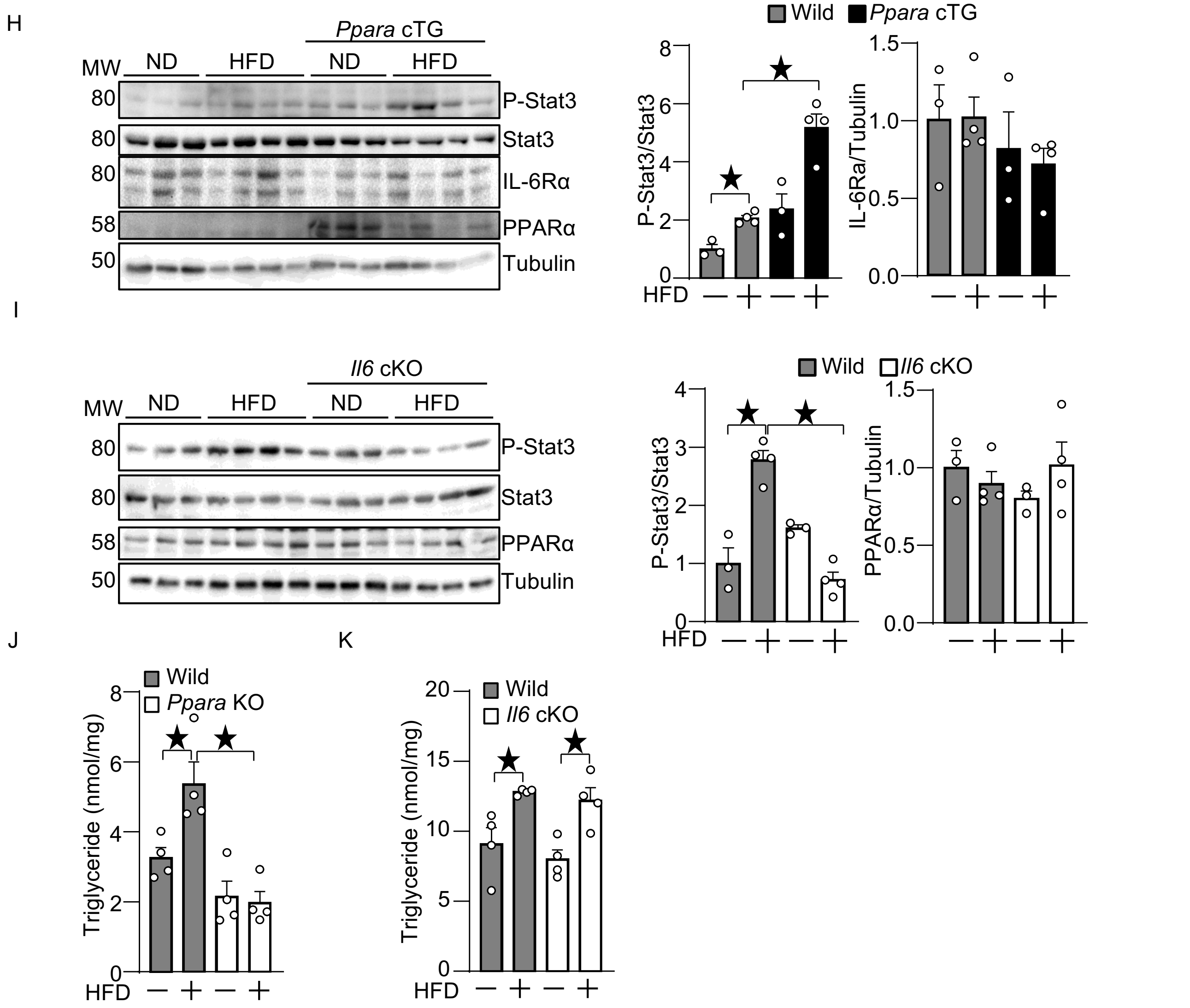


**Figure S3.** Significant immune cell infiltration into the heart was not observed after 1 month of HFD consumption. (A) The populations of the indicated cell types in the heart were examined with FACS analyses. (B) The mRNA levels of *Cd68* in the heart were examined in PPARα gene manipulated mice. (C-D) Macrophage depletion with clodronate did not affect HFD-induced diastolic dysfunction. Clodronate (5 mg/ml x 0.2 ml/head) was injected twice a week into the mice fed HFD. (C) Macrophage depletion was confirmed with a decrease in CD68 proteins in the heart. (D) Diastolic function was examined after 1 month of HFD consumption. (E) *Il6* mRNA levels in the heart after high-fat diet feeding in *Ppara* cKO mice, shown separately for male and female groups as well as combined data. (F) HFD significantly induced IL-6 expression in cardiomyocytes (CMs), but not in the non-cardiomyocyte (NCM) fraction. (G) HFD stimulated IL-6 signaling, characterized by phosphorylation of Stat3, in a PPARα-dependent manner. (H) Cardiac specific *Ppara* expression enhanced HFD-induced IL-6 signaling. (I) Cardiomyocyte *Il6* loss did not significantly affect PPARα expression. (J) HFD-induced increase in myocardial triglyceride content is PPARα-dependent. (K) IL-6 does not mediate lipotoxicity under HFD feeding conditions. The numbers of mice examined in each experimental group were: 3-6 (A), 7-8 (B), 3 (C), 6-7 (D), 8 (E), 4(F), 3-4 (G-I), and 4(J-K). Statistical significance is indicated by a star and was assessed using the Kruskal–Wallis test (B), Student's t test (C) and ANOVA for all other comparisons. Statistical analysis was not performed for females in (C) due to the insufficient sample size of the *Ppara* cKO ND group.

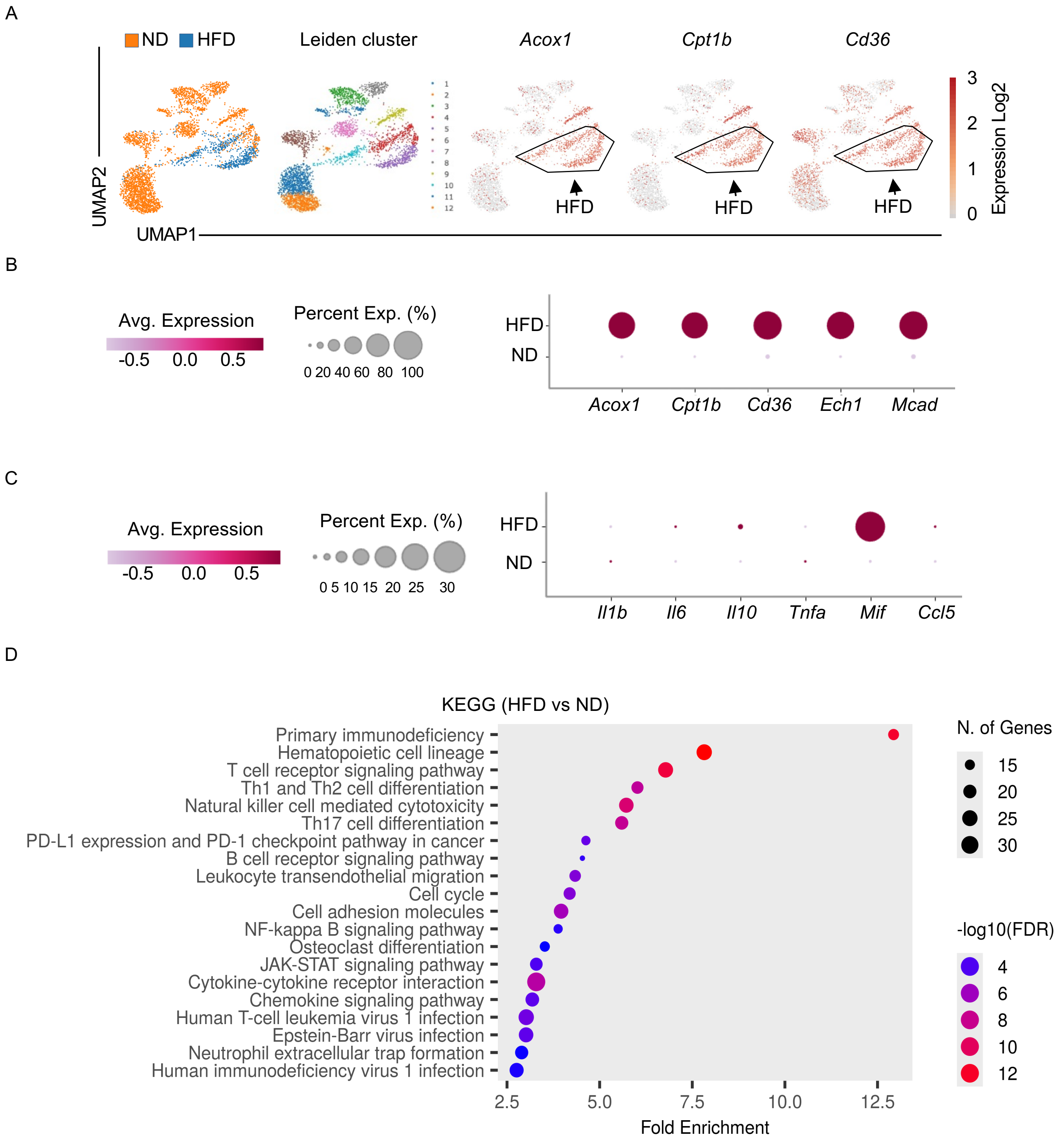


**Fig. S4** Single-cell RNA sequencing (scRNA-seq) shows upregulation of PPAR target genes and *Il6*in cardiomyocytes in response to HFD consumption. (A) Uniform manifold approximation and projection (UMAP) plots of cardiomyocytes from ND- and HFD-fed mice, showing cluster annotations and distribution of cells across diet conditions. Arrows highlight cluster shifts upon HFD exposure. UMAP plots indicate expression levels of PPAR target genes, including *Acox1*, *Cpt1b* and *Cd36*. (B-C) Dot plot visualization of PPAR target gene (B) and cytokine (C) expression from scRNA-seq data. (D) KEGG pathway enrichment analysis of differentially expressed genes (DEGs) between ND- and HFD-fed mice.

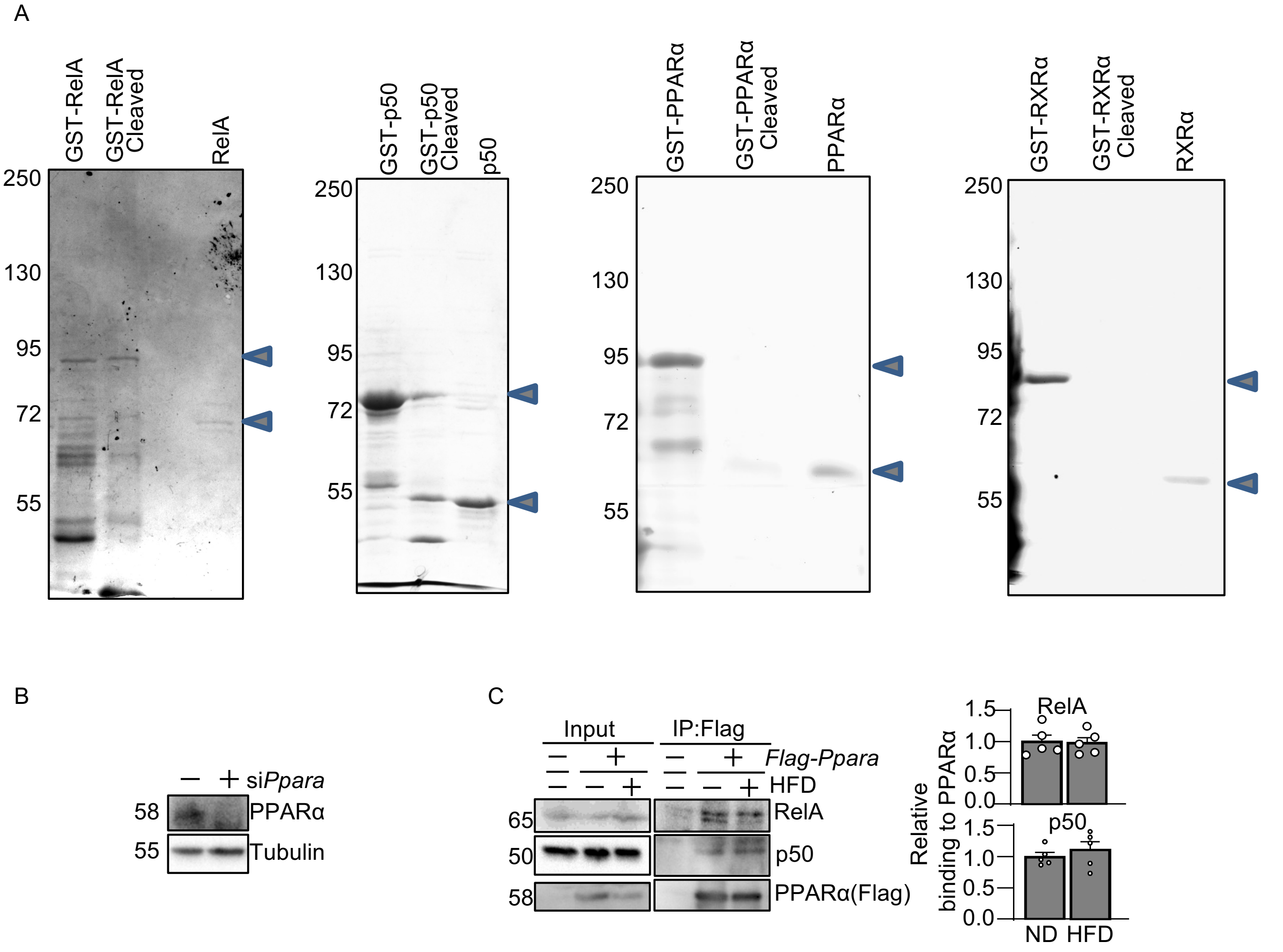


**Figure S5.** (A) Purity of recombinant proteins used by this study. GST-fused recombinant proteins were expressed and purified from *E.coli*. GST was cleaved by precision protease. The recombinant proteins were subjected to SDS-PAGE and CBB staining. (B) Cultured cardiomyocytes with *Ppara* knockdown were treated with 300 μM PA for 16 hours. Knockdown of PPARα was verified by Western blot analysis.
(C) PPARα binds to both RelA and p50 subunit in the heart. Flag-PPARα was immunoprecipitated from cardiac-specific *Flag-Ppara* transgenic mice. N=5. Statistical significance was assessed using Student's t-test.

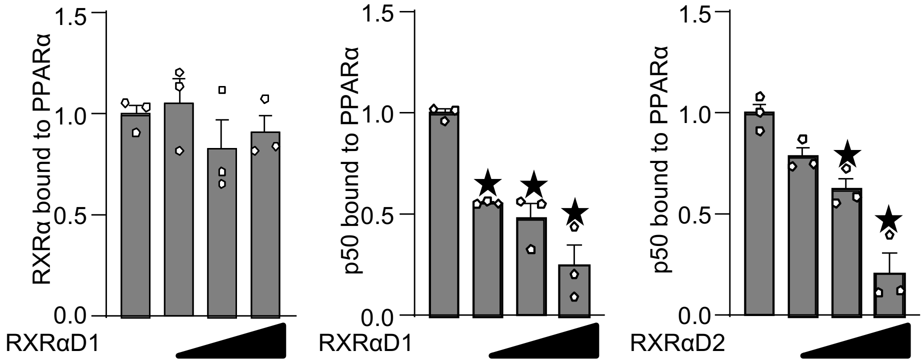


**Figure S6**. RXRαD1 inhibits the binding of PPARα to p50 but not to RXRα. Densitometric analyses were performed after *in vitro* binding assays. N=3. Statistical significance is indicated by a star and was assessed using ANOVA.
