# Supplementary material for: PPARα–NF-κB heterodimer mediates obesity-induced diastolic dysfunction through autocrine production of IL-6
Source: J Clin Invest. 2026 Feb 12;136(7):e196238. doi: 10.1172/JCI196238 (PMC13038213; doi:10.1172/JCI196238)

Unedited blot and gel images

Oka et al

Figure 3F

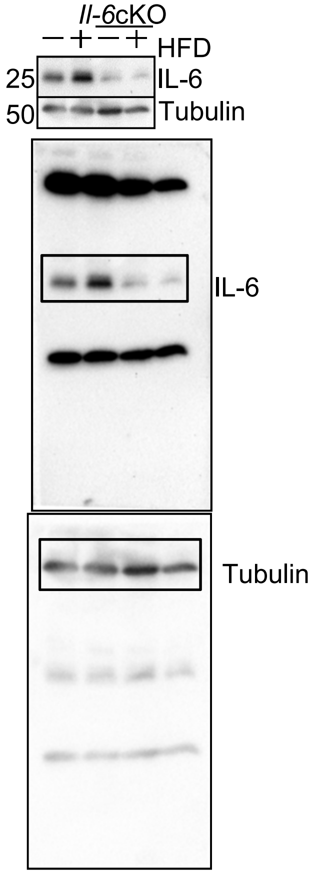

Figure 4D

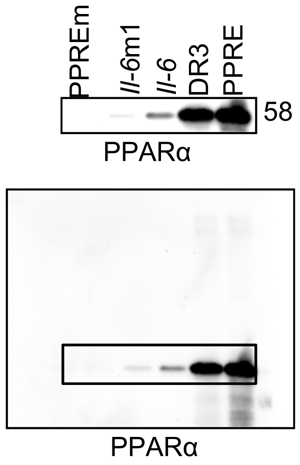

Figure 4J

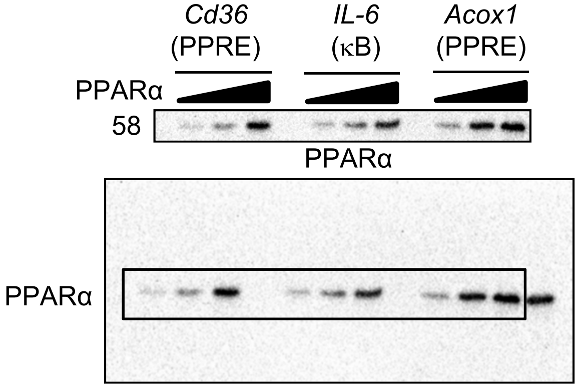

Figure 5A

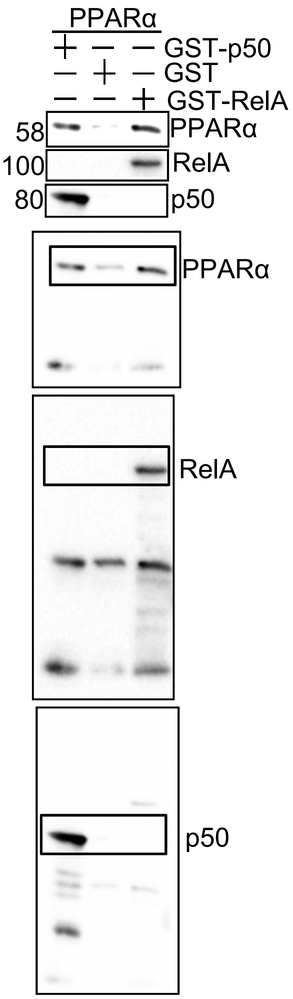

Figure 5B

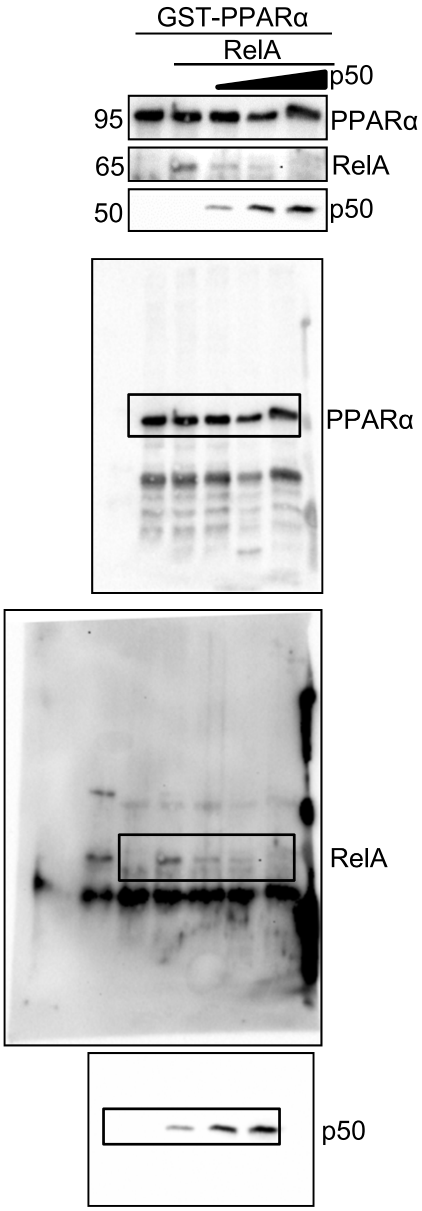

Figure 5C

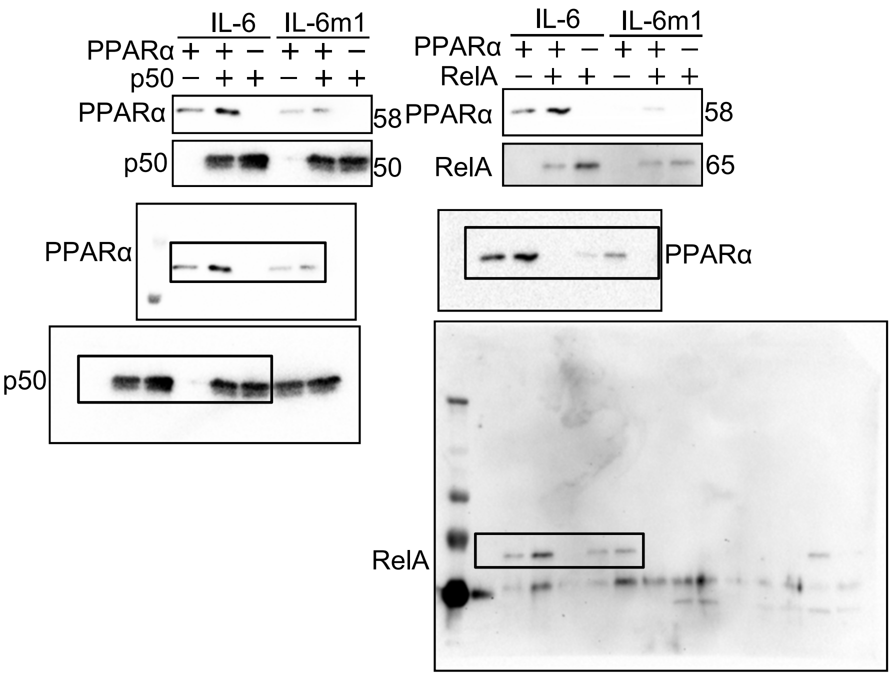

Figure 5D

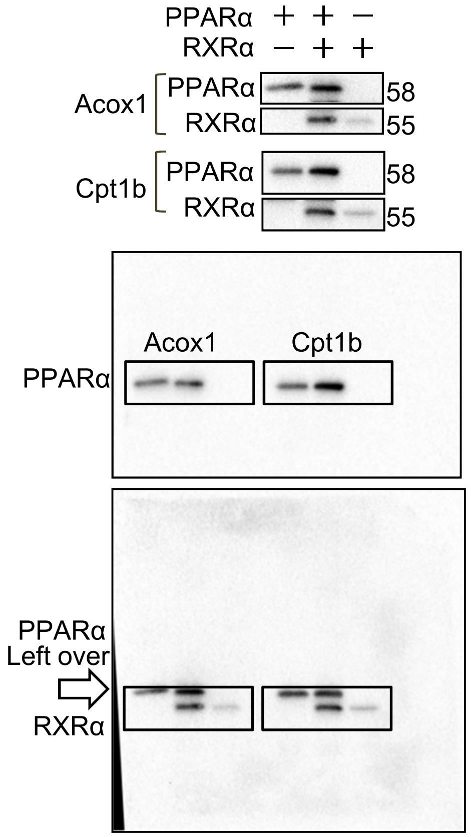

Figure 5G

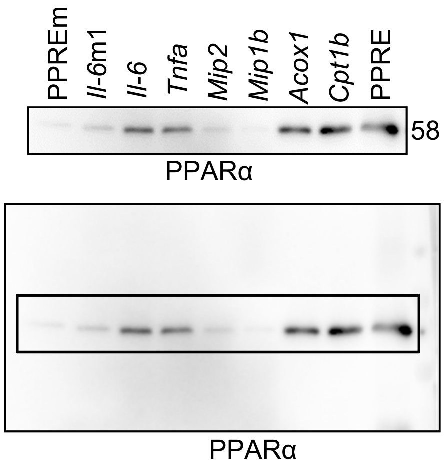

Figure 5H

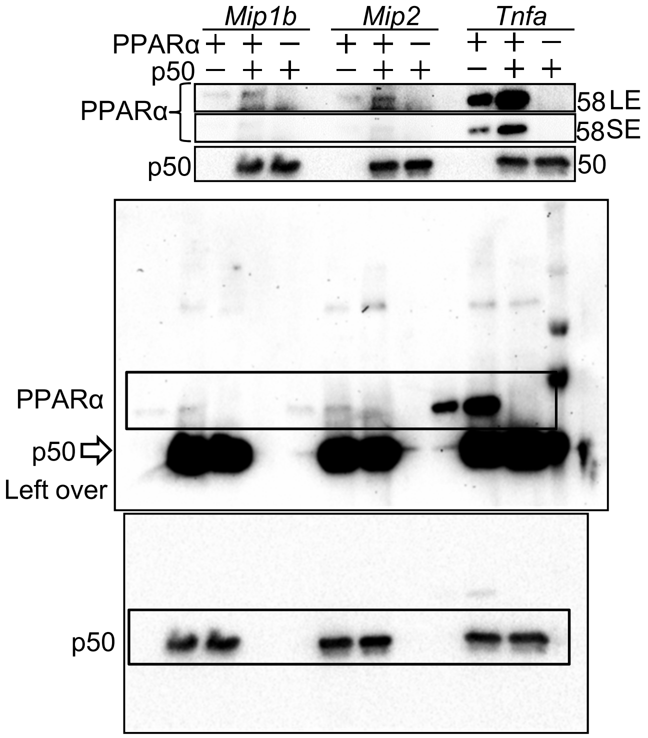

Figure 6B

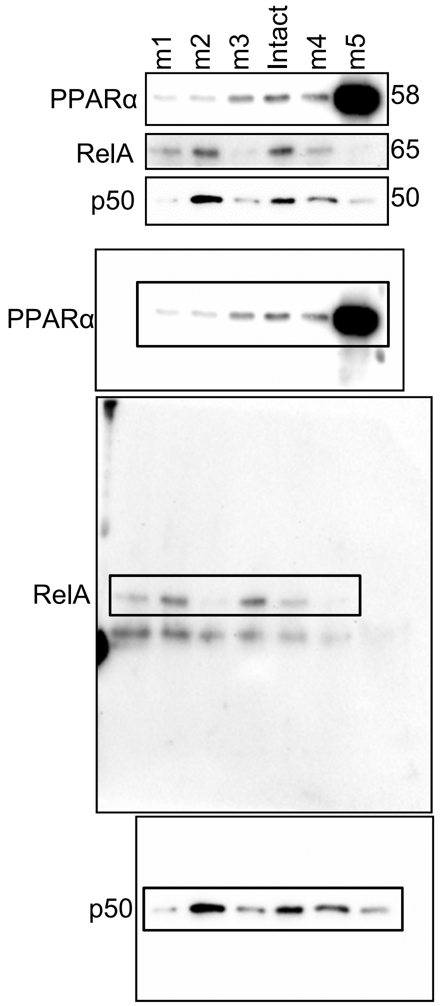

Figure 6C

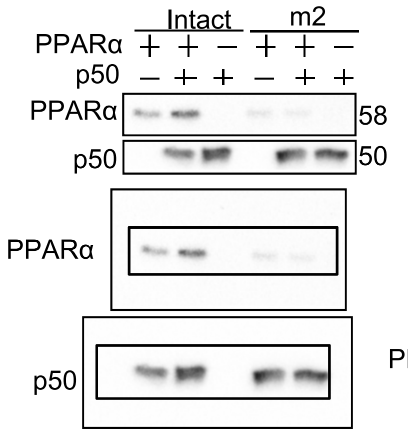

Figure 6D

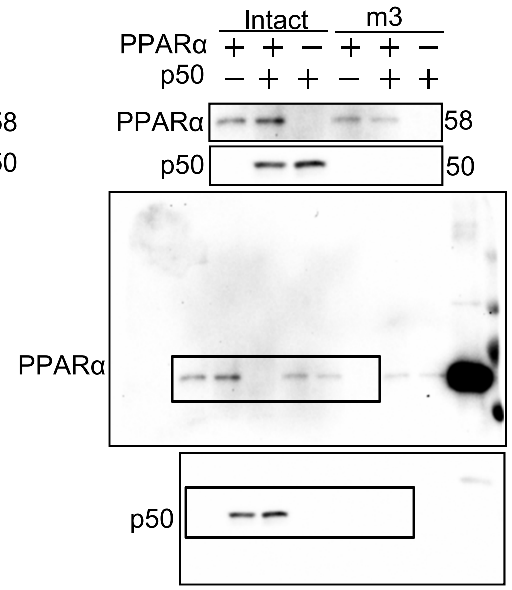

Figure 6E

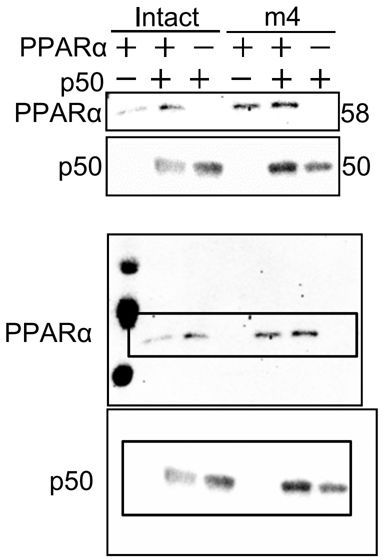

Figure 7B

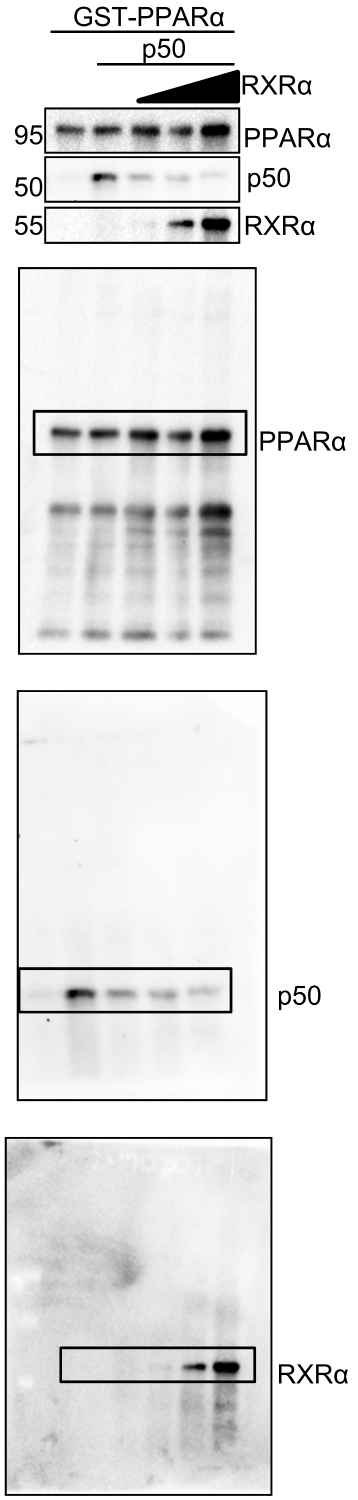

Figure 7C

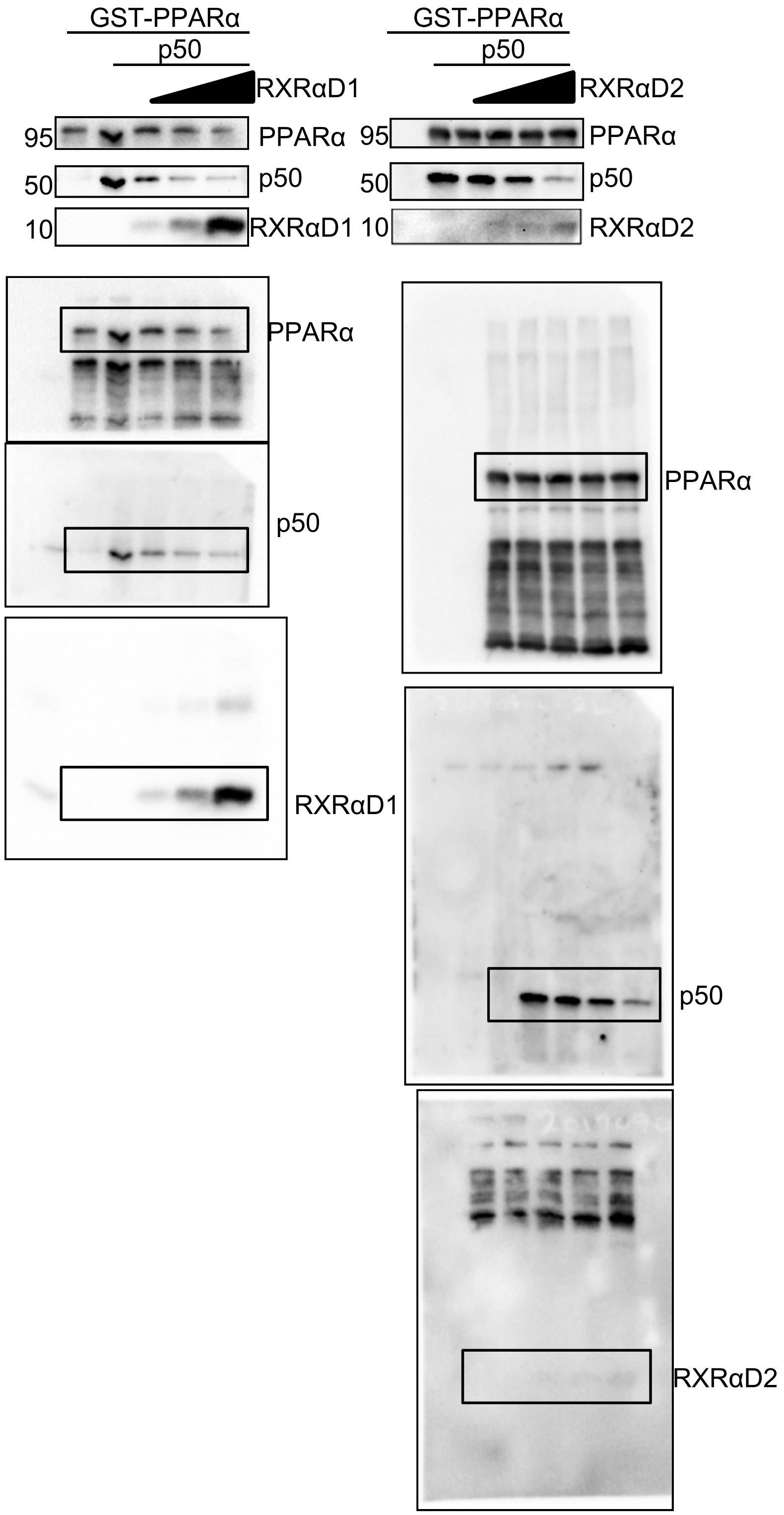

Figure 7D

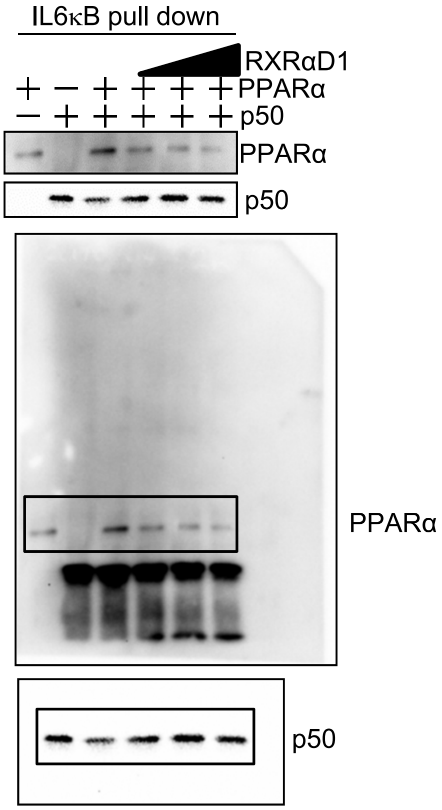

Figure S3C

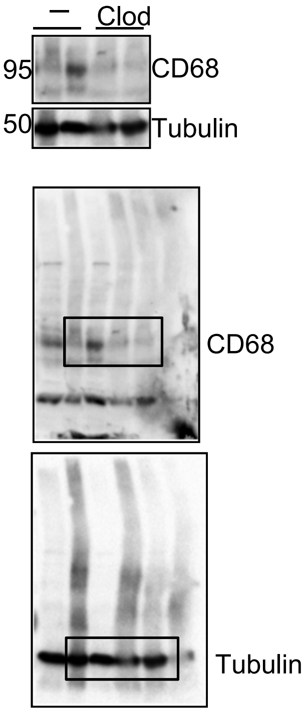

Figure S3F

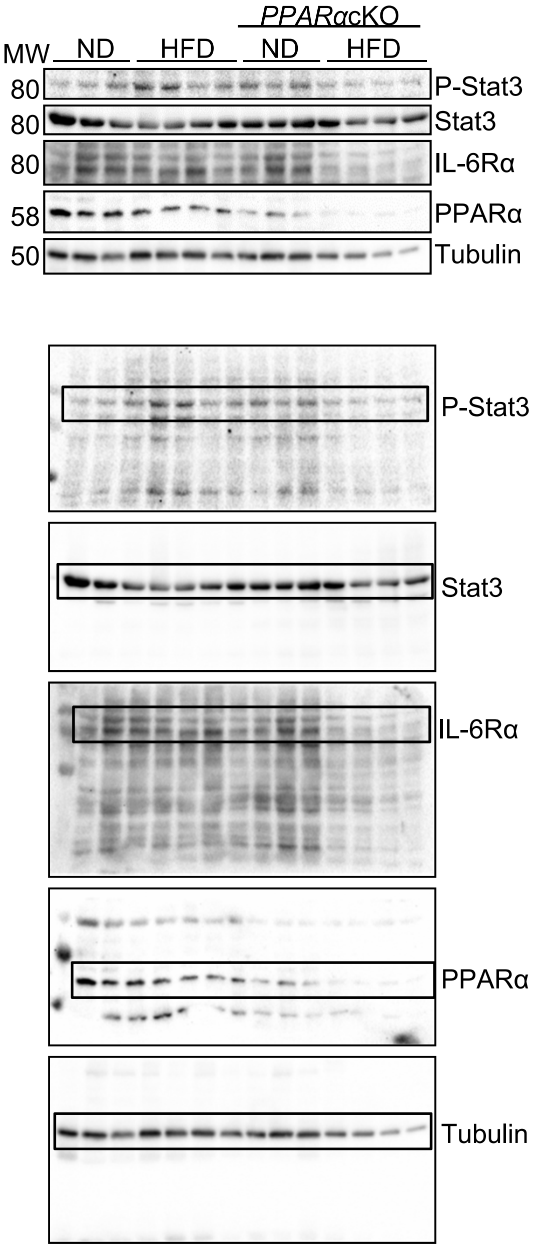

Figure S3G

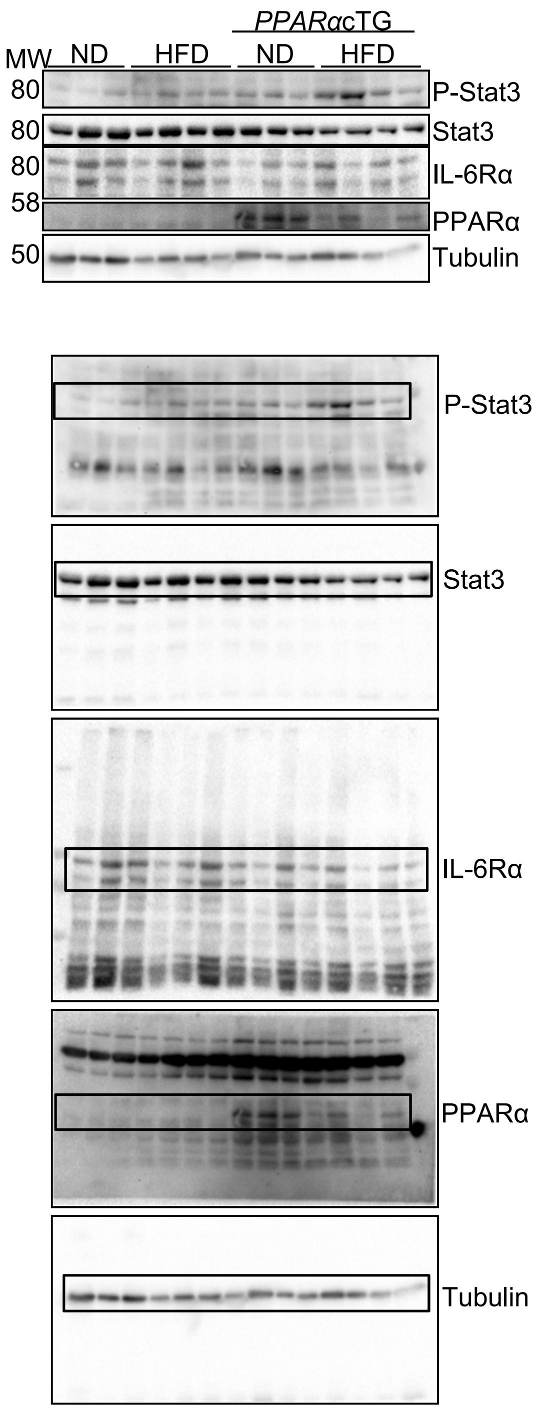

Figure S5B

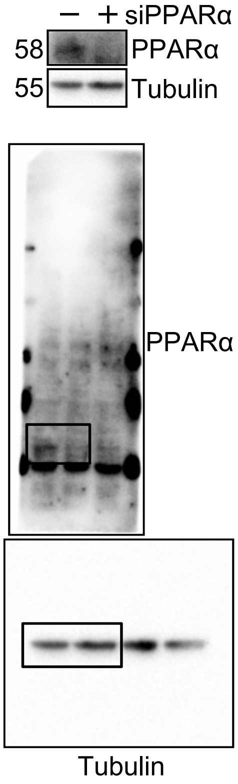

Figure S5C

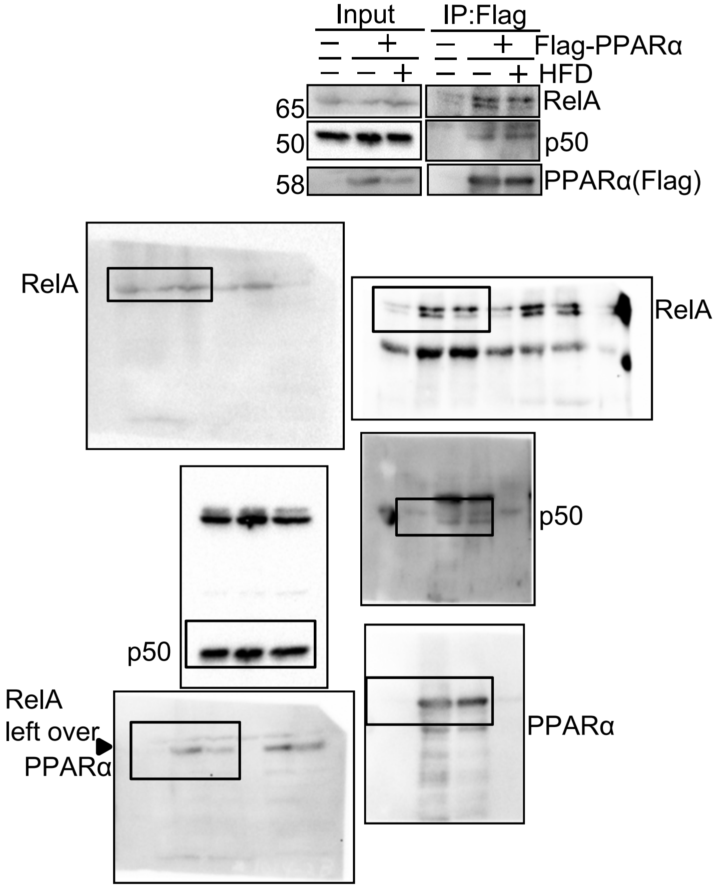

Supplement: Unedited blot and gel images [file jci-136-196238-s280.pdf]
